# Dairy Intake and Iodine Status in Pregnant and Lactating Women: A Systematic Review and Meta-Analysis

**DOI:** 10.3390/nu17233765

**Published:** 2025-11-30

**Authors:** Elham Razmpoosh, Katrina Geronimo, Caroline Gauntlett, Isabella Vicente, Patricia Pham, Aarsh Shah, Kathy Musa-Veloso

**Affiliations:** Intertek Health Sciences Inc., 2233 Argentia Road, Suite 201, Mississauga, ON L5N 2X7, Canada; elham.razmpoosh@intertek.com (E.R.); katrina.geronimo@intertek.com (K.G.); caroline.gauntlett@intertek.com (C.G.); isabella.vicente@intertek.com (I.V.); patricia.pham@intertek.com (P.P.); aarsh.shah@intertek.com (A.S.)

**Keywords:** iodine, dairy, pregnancy, lactation, milk, urinary iodine concentration, maternal nutrition

## Abstract

**Background/Objectives**: Despite salt iodization, iodine deficiency during pregnancy and lactation is re-emerging in many industrialized countries, necessitating an evaluation of the role of dairy in supporting iodine status during these critical periods. **Methods**: We conducted a systematic review and meta-analysis in accordance with PRISMA guidelines. Ten databases were searched through March 2025 using ProQuest Dialog™ (Version 75.0). Study quality was assessed using either the Quality Evaluation for Observational Studies tool or the Risk of Bias 2 tool, depending on study design. Random-effects models were applied, with certainty of evidence rated using the GRADE framework. Publication bias, sensitivity analyses, and subgroup analyses were also performed. **Results**: Fifty-one publications met the eligibility criteria, including 50 publications of observational studies and 1 publication of a randomized controlled trial (RCT), with most studies conducted on pregnant women. Higher dairy intake was associated with significantly greater urinary iodine concentration (UIC), (23 studies; standardized mean difference: 0.326; 95% confidence interval [CI]: 0.228, 0.424; *p* < 0.001; *I*^2^ = 57.31%; low-certainty), and significantly lower odds of iodine deficiency (11 studies; odds ratio: 0.58; 95% CI: 0.48, 0.70; *p* < 0.001; *I*^2^ = 0%; moderate-certainty). Associations were stronger in studies conducted during later pregnancy, in higher-quality studies, and when the dairy food was specifically milk. Dairy contributed to ~27% of iodine intake from foods. Breast milk iodine concentration (BMIC) findings were inconsistent, though in one RCT, iodine-fortified milk improved BMIC and UIC. **Conclusions**: Dairy intake supports adequate iodine status during pregnancy and lactation. RCTs would be valuable in further investigating the role of dairy in supporting iodine status, particularly in lactating women. (PROSPERO CRD420251054576)

## 1. Introduction

Iodine is an essential mineral for the production of thyroid hormones including thyroxine and tri-iodothyronine [1]. During pregnancy, maternal iodine requirements increase due to the transfer of thyroid hormones and iodine to the fetus, as well as greater maternal production of thyroid hormones [2,3,4]. Adequate iodine is especially important during early pregnancy when the fetus is entirely dependent on maternal thyroid hormones before its own thyroid becomes functional [1,5]. Mothers also have increased iodine requirements during lactation, as infants depend on the iodine secreted in breast milk; thus, sufficient iodine is required for normal thyroid function in both the mother and the infant during lactation [2,6].

Adequate thyroid hormones are important given their role in many aspects of fetal growth and development, particularly brain development. Thyroid hormones are required for neuronal proliferation and migration, glial differentiation, and myelination [5,6]. Moderate to severe iodine insufficiency during pregnancy has several consequences including a higher risk of congenital hypothyroidism, growth and developmental delays, stillbirth, miscarriage, and mortality [6,7]. There is some evidence that mild-to-moderate iodine insufficiency during pregnancy may also result in impaired cognitive function such as lower intelligence scores and impaired executive function during infancy and childhood [6,8].

The World Health Organization (WHO) recommends that pregnant and lactating women consume 250 µg of iodine per day [9]. Similarly, other authoritative bodies recommend total daily intakes of 200 to 220 µg of iodine per day for pregnant women and 200 to 290 µg of iodine per day for lactating women [10,11].

Universal salt iodization (USI) programs are the key strategy adopted in the elimination of iodine deficiency disorders, but despite the implementation of these programs in several countries, iodine deficiency in pregnant and lactating women has re-emerged as a global concern [9,12]. In a meta-analysis of observational studies, the overall prevalence of insufficient iodine intake in pregnant women was 53%, and prevalence was only marginally decreased to 51% when considering only countries with adequate or excessive iodine status [12].

One remediation strategy recommended by the WHO for countries or regions with lapsed or unequal distribution of USI programs is the consumption of an oral iodine supplement providing 150 µg of iodine per day [9]. Iodine intakes > 500 µg/day are generally not recommended, as there are no additional health benefits [9]. Indeed, a recent review of four meta-analyses of epidemiological studies suggested a U-shaped correlation between iodine status and several adverse maternal and fetal outcomes, indicating that both insufficient and excessive iodine intakes may have adverse effects [13]. In the same review, it was reported that in three meta-analyses of iodine intervention studies [14,15,16], conclusive evidence of the benefits of iodine supplementation in women in areas with mild-to-moderate iodine deficiency was not provided [13].

Given the limited evidence on the benefits of oral iodine supplementation, a focus on iodine intake from food sources to address iodine insufficiency is warranted. In addition to iodized salt, dietary sources of iodine include seafood, meat, eggs, bread, and dairy [17]. With shifts in dietary patterns and limited use of iodized salts in the production of processed foods, iodine deficiency is still prevalent in countries with otherwise strong public health systems [18]. Moreover, given the association of salt with hypertensive disorders of pregnancy [19], pregnant women may deliberately limit their sodium intake. Consumption of a plant-based diet may also contribute to iodine deficiency given that fish and dairy products such as milk are key iodine sources for pregnant women in many areas including Europe [17]. Furthermore, seafood consumption during pregnancy has declined due to public health guidelines recommending that pregnant women reduce their exposure to mercury [20].

The increase in iodine insufficiency may also be attributed to declining milk consumption. In the United States, the contribution of milk iodine to overall iodine intake decreased by 6.4% between 2011 and 2020 in women and girls [21]. This is especially concerning given the positive association between urinary iodine concentrations (UICs) and milk iodine intake [21]. However, it should be noted that iodine concentrations in milk across industrialized countries are widely variable, ranging from concentrations of 33 to 534 µg/L [22]. The wide variation is influenced by iodine concentrations, goitrogens, and iodine agonists in cow feed; the use of iodine supplements in cows or cow feed; regional milking practices, such as the use of iodinated sanitizers on udders prior to milking; and season [23].

Multiple systematic reviews on the determinants of iodine status have demonstrated that milk and dairy products are significant contributors to iodine intake [8,17]. However, to date, associations between dairy consumption and iodine status in pregnant and lactating women have not been systematically reviewed and meta-analyzed. Furthermore, it is unknown whether these associations are consistent across regions where cattle rearing and milking practices may differ substantially. Thus, the objectives of this systematic review and meta-analysis are to determine the relationship between dairy product intake and iodine status, as well as the contribution of dairy product intake to total iodine intake or status in pregnant and lactating women. To our knowledge, this is the first systematic review and meta-analysis to quantify these associations specifically in these populations.

## 2. Materials and Methods

### 2.1. Protocol

The study was conducted according to the Preferred Reporting Items for Systematic reviews and Meta-Analyses (PRISMA) 2020 guidelines [24]. The protocol for this systematic review and meta-analysis was registered in the International Prospective Register of Systematic Reviews (PROSPERO) on 2 June 2025 under registration number CRD420251054576.

### 2.2. Literature Search

#### 2.2.1. Databases and Literature Search Strategy

Ten literature databases (AdisInsight: Trials, Allied & Complementary Medicine™, BIOSIS Previews^®^, CAB ABSTRACTS, Embase^®^, Embase Preprints, Foodline^®^: SCIENCE, FSTA^®^, MEDLINE^®^, and NTIS: National Technical Information Service) were searched on 12 August 2024 and 5 March 2025 using the electronic search tool ProQuest Dialog™ (Version 75.0) (Clarivate, Philadelphia, PA, USA, 2022). Duplicates were automatically removed using ProQuest Dialog™ (Version 75.0). Keywords related to iodine, dairy, and pregnant or lactating populations were searched. For an article to be identified, the term “iodine” or “iodide” had to appear in the title, together with at least 1 term for the study population (e.g., “pregnant”). Additionally, at least 1 term for dairy (e.g., “milk”) had to appear either in the title or abstract of the article. No limitations were placed on the language of publication. The original search on 12 August 2024 had no limitations on the publication date, while the updated search on 5 March 2025 was limited to studies published on or after 12 August 2024. In addition to the studies identified through database searching, reference lists of recent systematic or narrative reviews were also screened to ensure the identification of all relevant studies. The date ranges and search strategy used to retrieve literature are further detailed in Supplementary Tables S1 and S2, respectively.

#### 2.2.2. Eligibility Criteria

Full-length articles published in peer-reviewed journals, publication preprints, or unpublished full study reports were eligible for inclusion. Studies in pregnant or lactating women of any age in which the iodine status (assessed through UIC, urinary iodine-to-creatinine ratio [I/Cr], breast milk iodine concentration [BMIC], or dietary intake) of the participants in relation to dairy consumption was assessed were included. The setting included observational studies (prospective, retrospective, and cross-sectional), as well as randomized controlled trials (RCTs). In addition, the study had to be related to the intake of dairy products (including milk, yogurt, cheese, cream, butter, and other dairy products, non-fermented or fermented) and the comparator in the study had to be individuals with low or no dairy intake.

Studies that met one or more of the following criteria were excluded: studies without a full study report; animal or in vitro studies; case series and case-control studies; studies in which iodine status and dairy consumption were not reported for pregnant/lactating women separately from other demographics; studies where subjects were taking iodine-containing medications or undergoing iodine therapy; studies focusing on isolated components of dairy, non-dietary iodine sources, or fortified or non-fortified foods other than dairy; studies in which dairy was reported in combination with other food groups or supplements and the independent effect or association of dairy was not reported; studies without a clear reporting of dairy intake; studies where iodine-related outcomes were not clearly reported; secondary research studies; and duplicate records in the literature.

#### 2.2.3. Literature Filtration

The eligibility criteria were applied by at least 2 independent reviewers (C.G., E.R., I.V., K.G., and/or P.P.) in 3 stages using the titles, abstracts, and full-length texts of the publications. Disagreements were resolved through discussion or consultation with a third reviewer (E.R., K.G., or K.M-V). Articles published in languages other than English were machine-translated to assess eligibility. Non-English articles that were considered eligible for inclusion based on the machine translation were formally translated.

### 2.3. Quality Assessment of Studies

The quality of all included studies was assessed in duplicate by 2 independent reviewers (C.G., I.V., K.G., P.P., and/or E.R.) and discrepancies were resolved with input from a third reviewer (K.M-V.).

The quality of the included observational studies was assessed using the Observational Study Quality Evaluation (OSQE) tool, which is suitable for the assessment of both cohort and cross-sectional studies [25]. The OSQE prospective study tool has a maximum score of 14 to 16 stars and has 14 obligatory items (representativeness of study population [2 stars]; validity of the independent variable [2 stars]; validity of the dependent variable [5 stars]; loss to follow-up [3 stars]; and miscellaneous, including control of relevant confounders in the statistical analysis [1 star] and conduct of analyses in accordance with methods defined a priori in a protocol [1 star]) and 2 optional items (effect modifiers [1 star]; and sample size if not meta-analyzing data [1 star]). The OSQE cross-sectional study tool has a maximum of 7 to 10 stars and has 7 obligatory items (representativeness of study population [1 star]; validity of the independent variable [2 stars]; validity of the dependent variable [1 star]; conflict of interest [1 star]; and miscellaneous, including control of relevant confounders in the statistical analysis [1 star] and conduct of analyses in accordance with methods defined a priori in a protocol [1 star]) and 3 optional items (methods to deal with missing data [1 star]; effect modifiers [1 star]; and sample size if not meta-analyzing data [1 star]).

In the present study, we considered all items except for the optional item “sample size if not meta-analyzing data,” as we pooled data from all included studies in a meta-analysis. Therefore, the maximum possible score was 15 for cohort studies and 9 for cross-sectional studies. After group consensus, we classified cohort studies with scores of ≤7/15, 8/15, and ≥9/15 as low, medium, and high quality, respectively. For cross-sectional studies, we classified scores of ≤3/9, 4/9 to 5/9, and ≥6/9 as low quality, medium quality, and high quality, respectively.

The Risk of Bias 2 (ROB2) tool was used to assess the quality of included RCTs [26]. Studies were assessed as having either low or high risk of bias or some concerns based on 5 domains related to the randomization process, deviations from the intended intervention, missing outcome data, measurement of the outcome, and selection of the reported result.

### 2.4. Data Extraction

All relevant study data were extracted independently by 1 reviewer, and the accuracy of the extracted data was verified by a second reviewer (E.R.). Any discrepancies were resolved with input from a third reviewer (K.M-V.). For all included studies, we extracted the first author’s name and year of publication, data related to the study design, country of conduct, data collection period, population characteristics (including sample size, age, stage of pregnancy or lactation, and thyroid disorder status), iodine-containing supplement use, iodized salt intake, and season. We also extracted details of the exposure (dairy intake, dairy food(s), and method of dietary assessment) and outcomes (biomarkers measured, iodine intake estimates, sampling method, and definition of iodine deficiency). Results were also extracted, including descriptive statistics for total, high, and low dairy intake groups for each iodine biomarker. Iodine intake measures (total and from dairy) were extracted together with the percentage contribution of dairy to total iodine intake and iodine status. We also extracted effect estimates (i.e., odds ratios [ORs], beta coefficients, correlations), covariates used if models were adjusted, comparison groups, confidence intervals (CIs), and *p*-values. Data for the highest vs. lowest dairy intake groups for each iodine-related outcome were extracted to enable consistent pooling. In studies reporting multiple effect estimates for the association between dairy intake and iodine status, the estimate from the model with the most complete adjustment for potential confounders was extracted.

Additional information was sought from study investigators if the required information was unclear or unavailable in the study publications.

### 2.5. Data Synthesis

A narrative synthesis of the findings from the studies was conducted according to the outcome measure.

For outcome measures with data from at least 2 studies, a meta-analysis was conducted comparing the highest vs. lowest categories of dairy intake. Continuous data were pooled using the generic inverse-variance method with DerSimonian and Laird random-effects models and expressed as standardized mean differences (SMDs) with 95% CIs. For SMDs, Cohen’s d was interpreted as follows: 0.2 represents a small effect, 0.5 a moderate effect, and 0.8 a large effect [27]. For event data, if studies did not report ORs, these were calculated in Comprehensive Meta-Analysis (CMA) software (Version 4.0.000) (Biostat, Englewood, NJ, USA, 2022) using event data provided in each study, and these ORs were considered “unadjusted” for the purposes of our sensitivity analyses. For the percent contribution of dairy to iodine intake, as measures of variability for this outcome were not provided in most of the studies, the weighted contribution was calculated using sample size as the weighting variable. For outcomes reported as beta coefficients or correlation coefficients, data were first converted to Fisher’s Z using established formulas [28,29], and then pooled separately.

For studies reporting results separately for different exposures (e.g., milk, yogurt, and cheese), we combined them unless a broader category (e.g., total dairy) was explicitly reported, in which case results for the broader category were used. In several studies, both adjusted and unadjusted results were reported. The most highly adjusted results were used for the main analysis; in the absence of adjusted results, the unadjusted results were used in the main analysis. In sensitivity analyses, adjusted and unadjusted results were separately pooled. If exposures and outcomes were assessed using multiple tools, those that were used in the majority of the studies were selected for inclusion in the main analysis to optimize consistency. If exposures and outcomes were assessed at different time points throughout pregnancy and/or lactation (e.g., first and second trimesters) or in different subgroups of women (e.g., prenatal supplement users and non-users), the average effect over all time points and subgroups was first calculated using a fixed-effects model and then used in the main analysis. To evaluate the robustness of the findings, as part of sensitivity analyses, we also separately pooled (i) unadjusted outcomes; and (ii) outcomes assessed using different methodologies (e.g., UIC and urinary iodine excretion [UIE]) or at different time points (e.g., first trimester, second trimester).

Random-effects models were used because no limits were placed on the region of study conduct or year of publication.

Heterogeneity across studies was assessed using the *I*^2^ statistic and the chi-square test. An *I*^2^ value > 50% was considered indicative of substantial heterogeneity (Cochrane, Chapter 10 [30]). Data permitting, subgroup analyses were conducted based on population type (pregnant vs. lactating women), country/continent of study conduct, trimester or postpartum stage (based on the population mean), thyroid disorder status and objectivity of assessment, quality of studies, iodine supplement use, iodized salt use, method of iodine status assessment, type of dairy product, type of dairy assessment tool, and year of study. Sensitivity analyses were performed by excluding studies at high risk of bias.

For main analyses with a minimum number of 10 studies, funnel plots were visually inspected to assess the potential for publication bias (Cochrane, Chapter 13 [30]). In addition, the trim-and-fill method of Duval and Tweedie was used to statistically assess small-study effects.

All analyses were conducted using CMA software (Version 4.0.000).

### 2.6. Grading of Evidence

We assessed the certainty of findings for each association using the Grading of Recommendations, Assessment, Development, and Evaluations (GRADE) framework [31], which categorizes certainty as high, moderate, low, or very low based on 5 criteria. These criteria include risk of bias (rated as none [if most studies had high OSQE], serious [if most studies had medium OSQE], or very serious [if most studies had low OSQE]); inconsistency (rated based on heterogeneity and ranked as none [*I*^2^ < 50%], serious [50% ≤ *I*^2^ < 75%], or very serious [*I*^2^ ≥ 75%]); indirectness, which considers the applicability of the evidence to the review question; imprecision (rated as none [narrow confidence intervals not crossing the line of no effect and adequate sample size], serious [wide confidence intervals crossing the line of no effect, indicating uncertainty in the direction of the association], or very serious [very wide confidence intervals crossing the line of no effect and indicating high uncertainty around the effect estimate]); and other considerations (e.g., publication bias, dose-response gradient, effect size, and specificity of effect). Two authors conducted the GRADE assessments independently, and any discrepancies were resolved by consensus. The final ratings were determined using GRADEpro online software (GRADE Working Group; https://www.gradepro.org/, accessed on 25 August 2025).

### 2.7. Protocol Deviations

There are three deviations from the protocol that was submitted to PROSPERO, all of which were made without consideration of the results of the studies.

First, the OSQE tool, which is listed on the Latitude website as a valid tool for appraising the bias of observational studies of various designs, was used to appraise observational study quality instead of the Newcastle-Ottawa Scale (NOS), which was the tool specified for use in the protocol registered on PROSPERO. This is because the NOS was found to lack validity for appraising the risk of bias of cross-sectional studies.

Second, based on the protocol that was registered with PROSPERO, studies that included women with thyroid disorders were to be excluded; however, these studies were subsequently included because in many studies, no information on thyroid disorder status was provided, making it impossible to determine whether participants had such conditions. Excluding these studies would have resulted in the loss of a substantial number of otherwise eligible studies. To account for potential bias, we downgraded the quality rating for each study based on thyroid disorder status reporting, with studies that assessed thyroid status using laboratory tests and that excluded participants with thyroid disorders assigned a higher score.

Finally, the association of dairy with BMIC has been included as a secondary outcome, but was not pre-specified in the PROSPERO registration.

## 3. Results

### 3.1. Literature Search and Study Characteristics

**Figure 1:**
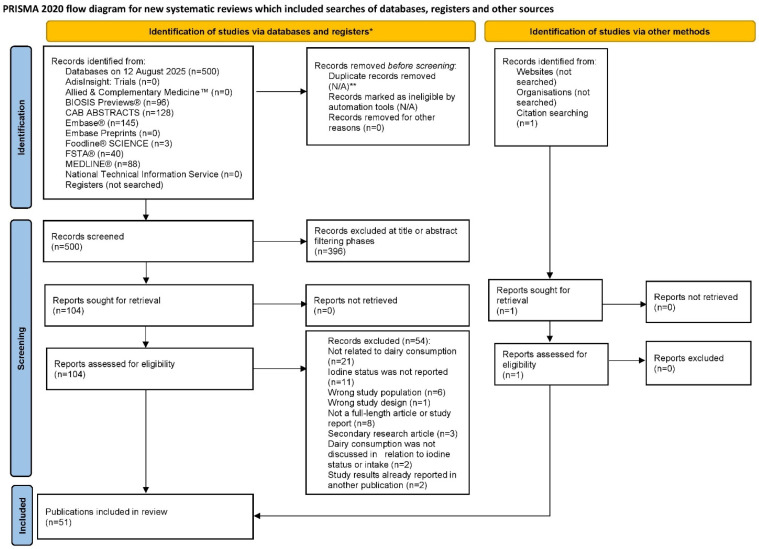
PRISMA flowchart of the literature search [24]. N/A = not applicable; PRISMA = Preferred Reporting Items for Systematic reviews and Meta-Analyses. * Source: Page et al. BMJ 2021;372:n71. doi: 10.1136/bmj.n71 [24]. This work is licensed under CC BY 4.0. To view a copy of this license, visit https://creativecommons.org/licenses/by/4.0/. ** Duplicates across databases were automatically removed by ProQuest Dialog™ (Version 75.0).

A total of 500 publications were identified through database searching and 1 additional publication was identified through citation searching of relevant review articles. Of the 501 publications identified in the literature search, 51 were found to meet all eligibility criteria [32,33,34,35,36,37,38,39,40,41,42,43,44,45,46,47,48,49,50,51,52,53,54,55,56,57,58,59,60,61,62,63,64,65,66,67,68,69,70,71,72,73,74,75,76,77,78,79,80,81,82]. Of note, 2 of the included studies were non-English publications that were translated into English [77,82]. A flow diagram of the literature search is provided in Figure 1.

### 3.2. Overview of Included Studies

Of the 51 included publications, 1 publication reported results from 1 RCT [67] and 50 publications reported results from observational studies. The single RCT [67], which was conducted in lactating women, was not included in the meta-analysis and is described in detail in Section 3.4.6.

Dineva et al. [46] reported results from 3 observational studies (Avon Longitudinal Study of Parents and Children [ALSPAC]; Generation R cohort; INfancia y Medio Ambiente [INMA]), and the publication by Castilla et al. [42] is a subgroup analysis of the INMA study. Also, González-Martínez et al. [49,50] reported results for the same study population but at different follow-up periods. In their 2009 publication, Brantsaeter et al. [39] reported on the validation of a food frequency questionnaire (FFQ) using a subset of the cohort, whereas in their 2013 publication, Brantsaeter et al. [40] used the validated FFQ in the entire cohort. In total, across the 50 observational study publications, the number of unique cohorts studied was 49.

Of the 49 observational study cohorts, 38 comprised pregnant women, 8 comprised lactating women [35,52,54,57,66,71,72,79], 1 comprised both pregnant and lactating women [75], and 2 comprised women who were pregnant at baseline but were followed both during pregnancy and lactation [34,76]. Among the observational studies, data were reported cross-sectionally for 42 cohorts, prospectively for 5 cohorts [32,42,44,62,76], and both cross-sectionally and prospectively for 2 cohorts [36,49,50]. Of the prospective analyses, 5 were conducted in pregnant women, while Aakre et al. [32] and Threapleton et al. [76] recruited participants during pregnancy and conducted multiple assessments throughout pregnancy and the postpartum lactation period. Nine of the 11 studies in lactating women reported data cross-sectionally. Studies of pregnant women included 25 to 61,904 participants with mean maternal ages between 22.6 and 34.1 years, while studies of lactating women included 50 to 928 participants with mean maternal ages between 28 and 34 years.

Of the 49 observational study cohorts, 30 were from 13 different countries across Europe: Spain (*n* = 7) [34,42,46,49,50,63,64,68,78], the United Kingdom (*n* = 6) [36,37,46,59,62,76], Norway (*n* = 5) [32,39,40,45,52,56], Iceland (*n* = 3) [33,51,71], Belgium (*n* = 1) [80], Cyprus (*n* = 1) [41], Denmark (*n* = 1) [55], Italy (*n* = 1) [65], Latvia (*n* = 1) [81], the Netherlands (*n* = 1) [46], Portugal (*n* = 1) [48], Sweden (*n* = 1) [75], and Croatia (*n* = 1) [72]. Eight of the cohorts were from Asia, including China (*n* = 3) [61,77,82], India (*n* = 2) [47,60], Taiwan (*n* = 2) [53,54], and South Korea (*n* = 1) [66]. Five cohorts were from Australia [35,38,43,44,57], 3 from the Americas (Brazil [*n* = 1] [74], Chile [*n* = 1] [69], United States [*n* = 1]) [70], 1 from Africa (Ethiopia [*n* = 1]) [58], and 2 from the Middle East (Saudi Arabia [*n* = 2] [73,79]). Dates of data collection, which were reported in 47 of the 50 observational study publications, ranged from 1991 to 2023, with seasonality additionally reported in 25 publications. Dairy intake was assessed using an FFQ in 32 studies, using a 24-h dietary recall in 5 studies [66,69,70,74,76], a general questionnaire in 6 studies [49,50,72,77,80,81], a structured dietary recall in 1 study [64], and a structured food checklist in another study [57]. A structured food questionnaire was used in 1 study [38], and Ollero et al. [68] used an iodine consumption questionnaire. In addition, Henjum et al. [52] used both an FFQ and a 24-h recall, and Brantsaeter et al. [39] used both an FFQ and food diary. Liu et al. [61] did not specify the type of dietary assessment tool used. A questionnaire validated in pregnant populations was used in 5 cohorts [33,39,40,42,45,46,78].

Dietary intake assessment periods varied across the observational studies. Usual intake was assessed in 30 of the observational studies. Intake specifically during pregnancy or shortly before pregnancy (previous week to first 5 months of pregnancy) was assessed in 11 studies [32,34,39,40,43,45,51,56,72,75,77]. Short-term intake (intake over the previous 24 h) was assessed in 4 studies [66,69,70,76]. In the Dineva et al. [46] study, usual intakes were assessed in the ALSPAC and INMA cohorts, whereas intake over the past 3 months was assessed in the Generation R cohort. Henjum et al. [52] provided data for both 24-h and usual intakes, intakes during pregnancy as well as in the last 24 h were reported in 2 studies [33,55], and in 1 study, the period of dietary assessment was not detailed [61].

Dairy products were categorized as “all dairy” (i.e., combined intakes of milk, cheese, yogurt, and cream) or by specific product types (milk, yogurt, cheese, or butter), with several studies reporting on multiple categories. Across the observational studies, “all dairy” intake was reported in 37 studies, while milk, yogurt, cheese, and butter intakes were reported in 25 studies, 10 studies, 8 studies, and 1 study, respectively. In addition, in 1 study, there was separate reporting of cheese consumption and of the combined consumption of milk and yogurt [72]. Across the observational studies, milk and dairy products were not reported as being directly fortified with iodine. In some studies, it was explicitly noted that iodine was added as a fortificant to cattle feed in that country, including Norway (7 publications [32,39,40,45,52,55,56]), Latvia (1 publication [48]), and Portugal (1 publication [81]).

Thyroid health was clinically investigated in 9 cohorts [49,50,54,59,61,63,69,72,73,76], and participants with thyroid disorders were explicitly excluded in 5 of these studies [54,59,61,69,72]. Key characteristics of the observational studies conducted in pregnant women and lactating women are provided in Supplementary Tables S3 and S4, respectively. The 1 study in which both pregnant and lactating women were recruited [75] and the 2 studies in which the women were pregnant at baseline but followed up with into the postpartum lactation period [32,76] are captured in Supplementary Table S3.

### 3.3. Results of Observational Study Quality Evaluations

Based on the OSQE tool, of the 43 cross-sectional studies, 3 were rated as high-quality studies [36,69,78], 29 as medium-quality studies, and 11 as low-quality studies [43,52,56,65,71,72,74,75,77,79,82]. Among the 5 prospective cohort studies, 2 were rated as high-quality studies [42,76], 1 as a medium-quality study [32], and 2 as low-quality studies [44,62]. Bath et al. [36] was assessed as both a cross-sectional study and a prospective cohort study; both study designs were identified as high-quality studies. Two studies by González-Martínez et al. were assessed as medium-quality, one a prospective cohort study [49] and the other a cross-sectional study [50]. Study quality is summarized in Supplementary Table S5.

### 3.4. Meta-Analysis

#### 3.4.1. Urinary Iodine Status Based on Dairy Intake

The association between dairy intake and urinary iodine status in pregnant or lactating women was assessed in 32 cross-sectional studies [33,34,36,37,38,39,41,45,46,48,50,51,52,53,55,56,58,59,61,64,65,68,69,70,71,72,73,75,77,78,79,81] and 2 prospective studies [42,62].

Data from Castilla et al. [42] were derived from the INMA cohort, which overlapped with data reported by Dineva et al. [46]. Because Dineva et al. [46] provided results from 3 cohorts (INMA, Generation R, and ALSPAC), the Castilla et al. [42] data were excluded to avoid duplication and the inflation of the results from the INMA cohort.

In several studies, multiple endpoints were reported and decisions had to be made about which endpoints to include in the meta-analysis on the association between dairy consumption and UIC. In all cases, decisions were made to maximize consistency between studies included in the meta-analysis. A synopsis of the decisions made is presented below.

In the study by Adalsteinsdottir et al. [33], dairy intake was assessed using FFQs covering the previous 3 months (reported by quartiles or as binary data—intake vs. no intake) and also using a 1-day FFQ; the quartiles derived from the 3-month FFQ were used in the main analysis. Two studies reported data separately for supplement consumers, non-consumers, and the overall population [34,48]; data for the overall population were included in the meta-analysis. Bath et al. [37] reported urinary iodine status separately according to milk intake and according to the intake of dairy products other than milk; for the latter, the numbers of women in the high and low intake groups were not reported, and so only the results for milk intake were included in the pooled analysis. In contrast, in another 3 studies in which milk intakes and dairy intakes excluding milk were separately reported, the results were sufficient for merging, thereby permitting a single average value for inclusion in the meta-analysis [36,38,41]. In 4 studies in which results were reported separately for specific dairy subtypes (e.g., milk, yogurt, cheese) [48,55,59,62], the relevant data were combined, and a single averaged estimate for dairy intake was used in the meta-analysis. In the study by McMullan et al. [62], intakes of milk and yogurt were presented using intake distributions (quintiles for milk; quartiles for yogurt), as well as using binary data (i.e., cut-offs to distinguish greater vs. lower consumption); the binary data were included in the meta-analysis. Finally, 5 studies reported urinary iodine status using both UIC and I/Cr [33,45,48,51,69]; UIC was used in the meta-analysis.

##### Urinary Iodine Status Based on Dairy Intake (Standardized Mean Difference)

**Figure 2:**
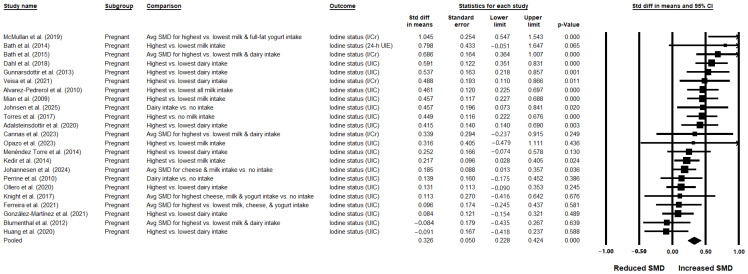
Meta-analysis of dairy intake and urinary iodine status using a random-effects model in pregnant women (studies in lactating women not identified) (*n* = 23 publications) [33,34,36,37,38,41,45,48,50,51,53,55,56,58,59,62,64,65,68,69,70,78,81]. In this forest plot, each study or stratum is represented by a square indicating the point estimate, with horizontal lines showing the 95% CI. The square size reflects the relative weight of the study or stratum in the analysis. The diamond reflects the pooled effect and its 95% CI. If a study reported UIC as well as other measures of urinary iodine, UIC was used in the meta-analysis, as it was the most commonly reported outcome across the studies. Avg = average; CI = confidence interval; h = hour; I/Cr = iodine-to-creatinine ratio; SMD = standardized mean difference; Std diff = standardized difference; UIC = urinary iodine concentration; UIE = urinary iodine excretion.

Mean and SD data on urinary iodine status for the highest (*n* = 3923) vs. lowest (*n* = 2927) categories of dairy intake were available from 23 studies [33,34,36,37,38,41,45,48,50,51,53,55,56,58,59,62,64,65,68,69,70,78,81] (all conducted in pregnant women) and were included in the pooled analysis. When data from all studies were pooled, urinary iodine status was significantly greater in the highest compared with the lowest dairy intake groups (SMD: 0.326; 95% CI: 0.228, 0.424; *p* < 0.001; *I*^2^ = 57.313%), corresponding to a small-to-moderate effect size with substantial heterogeneity (Table 1 and Figure 2). The overall certainty of the evidence was rated as low (Supplementary Table S6). Leave-one-out sensitivity analysis confirmed the robustness of the summary effect estimate (Supplementary Figure S1). Publication bias was not detected, and the trim-and-fill method did not impute any missing studies (Supplementary Figure S2). Only 1 prospective study was identified among the 23 included [62]; this study was an outlier in the main analysis (showing the greatest SMD for UIC in the highest vs. lowest dairy consumers). Excluding this prospective observational study did not alter the overall findings, though heterogeneity was slightly reduced (22 studies; SMD: 0.306; 95% CI: 0.214, 0.397; *p* < 0.001; *I*^2^ = 51.22%).

Sources of heterogeneity were investigated in the subgroup analyses. Though the numbers of studies were limited, subgroup analyses demonstrated statistically significantly greater urinary iodine levels in the highest vs. lowest dairy consumers with zero heterogeneity (*I*^2^ = 0%) when pooling studies conducted in Eastern European populations (2 studies [41,81]; SMD: 0.435; 95% CI: 0.05, 0.82; *p* = 0.027), studies rated as high quality (3 studies [36,69,78]; SMD: 0.525; 95% CI: 0.265, 0.785; *p* < 0.001), and studies conducted during the summer (4 studies [37,41,58,64]; SMD: 0.299; 95% CI: 0.022, 0.576; *p* = 0.034). Further subgroup analyses showed significant associations with lower heterogeneity when pooling studies of pregnant women in their third trimester (6 studies [34,45,58,59,65,69]; SMD: 0.390; 95% CI: 0.196, 0.585; *p* < 0.001; *I*^2^ = 35.08%). Sensitivity analyses restricted to studies of pregnant women who either used or did not use iodine supplements also confirmed greater urinary iodine levels in the highest vs. lowest dairy intake groups, with reduced heterogeneity in each analysis. The effect size was small-to-moderate among non-supplement consumers (2 studies; SMD: 0.396; 95% CI: 0.05, 0.742; *p* = 0.025; *I*^2^ = 0.0%), and moderate among supplement consumers (2 studies; SMD: 0.508; 95% CI: 0.027, 0.988; *p* = 0.039; *I*^2^ = 34.493%). Other sources of heterogeneity that were explored included thyroid status of participants (i.e., exclusion based on laboratory tests), dietary assessment tool, type of urinary iodine measure (I/Cr), and laboratory technique for urinary iodine assessment (high-performance liquid chromatography [HPLC]). Although limited data precluded a robust assessment of these variables as potential sources of heterogeneity, there were 5 studies in which iodine in urine was expressed as both UIC and I/Cr [33,45,48,51,69]. For these 5 studies, UIC was used in the main analysis to maximize consistency with the other studies; however, the overall analysis was also rerun using the I/Cr data instead of UIC from these 5 studies in order to assess the robustness of the findings. The association remained statistically significant but with higher heterogeneity between the studies (23 studies; SMD: 0.355; 95% CI: 0.236, 0.474; *p* < 0.001; *I*^2^ = 71.38%) (Supplementary Figure S3).

In 6 studies, associations of UIC with intakes specifically of milk were investigated [34,37,58,65,69,78]. Milk was used as a proxy for overall dairy intake for these 6 studies, and they were pooled with the other 17 studies in the main analysis for overall dairy intake. In a subgroup analysis restricted to these 6 studies, there was a significant positive small-to-medium association between milk intake and urinary iodine status, with markedly reduced heterogeneity (SMD: 0.405; 95% CI: 0.212, 0.598; *p* < 0.001; *I*^2^ = 1.393%) (Table 1). In an additional 7 studies, associations of UIC with milk intake were reported separately from associations with intakes of overall dairy or other dairy types (e.g., yogurt or cheese). When the 13 studies in which the association between milk intake and UIC were pooled, UIC was found to be significantly greater with higher vs. lower milk intakes, with substantial heterogeneity (SMD: 0.420; 95% CI: 0.268, 0.571; *p* < 0.001; *I*^2^ = 63.457%) (Table 1).

For studies with insufficient data for inclusion in the meta-analyses, most still supported the positive association observed in the meta-analysis. Bath et al. [37] reported greater 24-h UIE in the highest dairy tertile (median [interquartile range (IQR)]: 173.6 [121.3–203.0] µg/day) compared with the lowest tertile (mean [IQR]: 139.1 [85.3–203.9] µg/day]. In pregnant populations, Zhao et al. [77] reported lower urinary iodine levels among women who never or occasionally consumed milk or yogurt compared with those consuming at least 2 bottles per day. Three studies included lactating women [71,72,79]. Trabzuni et al. [79] reported that dairy intake had the most significant effect on urinary I/Cr in lactating mothers (*p* = 0.0008). Petersen et al. [71] similarly observed higher median UIC in lactating women consuming ≥2 portions/day of dairy compared with those consuming <2 portions/day. By contrast, Prpić et al. [72] found no significant association between milk, yogurt, or cheese intake and UIC in lactating women, though positive correlations between milk and yogurt consumption and breast milk iodine levels were reported (reviewed further in Section 3.4.3).

Considering seasonality, Bath et al. [36] reported a significant interaction between milk intake and season on I/Cr (*p* = 0.0003) among pregnant women. In those consuming >280 mL of milk/day, I/Cr was 1.27-fold higher in winter than in summer, highlighting a seasonal effect.

##### Urinary Iodine Status Based on Dairy Intake (Β Coefficients and Correlations, Converted to Fisher’s Z)

Among the 32 studies in which the association between dairy intake and urinary iodine status in pregnant or lactating women was examined, 6 studies [45,46,55,56,70,73] reported beta coefficients, 1 study [75] reported a correlation coefficient, and 1 study reported both a beta coefficient and a correlation coefficient [39]. One of these studies contributed results from 3 independent cohorts (ALSPAC, Generation R, and INMA) [46], resulting in data for a total of 10 unique cohorts. We converted all data to Fisher’s Z measures and pooled them in the meta-analysis. Among these, I/Cr was reported in 1 study [46], 24-h UIE was reported in 1 study [39], and 24-h UIC was reported in another study [73]. Pooling the results from all 8 studies and 10 cohorts yielded a significant small-to-moderate effect (SMD: 0.228; 95% CI: 0.154, 0.301; *p* < 0.001; *I*^2^ = 59.495%), though heterogeneity was substantial (Supplementary Table S7 and Supplementary Figure S4). The overall certainty of evidence was rated as low (Supplementary Table S6). The trim-and-fill method imputed 1 study missing to the left of the pooled effect; with this study imputed, the SMD remained significant in favor of a greater UIC with greater dairy intake (SMD: 0.226; 95% CI: 0.143, 0.292) (Supplementary Figure S5). Leave-one-out sensitivity analysis confirmed the robustness of the summary effect estimate (Supplementary Figure S6). After excluding the outlier study by Johanessen et al. [55], in which the smallest effect size was reported, heterogeneity decreased (*I*^2^ = 48.75%) and the effect estimate remained significantly positive (7 studies and 9 cohorts; SMD: 0.248, 95% CI: 0.18, 0.316; *p* < 0.001). Additionally, there was a minor decrease in heterogeneity when analyses were limited to studies reporting adjusted measures; the effect estimate remained significantly positive (7 cohorts [39,45,46,56,70]; SMD: 0.243; 95% CI: 0.145, 0.340; *p* < 0.001; *I*^2^ = 50.667%) (Supplementary Table S7).

#### 3.4.2. Iodine Deficiency Based on Dairy Intake

Sixteen studies reported on iodine deficiency in relation to dairy intake among pregnant or lactating women [32,34,40,47,48,49,50,54,58,60,63,65,77,78,80,81]. Of these, 12 assessed urinary iodine deficiency [32,34,48,49,50,54,58,65,77,78,80,81], and 2 provided dietary data on iodine deficiency comparing the highest vs. lowest dairy consumers [40,63]. The remaining 2 studies did not provide data suitable for pooling [47,60].

##### Urinary Iodine Deficiency Based on Dairy Intake

González-Martinez et al. [50] and González-Martinez et al. [49] studied the same cohort, with the 2021 report providing first trimester data and the 2023 report providing second trimester data. We merged these 2 reports, averaging data across the trimesters, and included the two studies as a single study in the final meta-analysis. Thus, data were available for 11 independent cohorts. Of these, 1 study was conducted among lactating women [53]. All studies assessed urinary iodine deficiency based on UIC, except for 1 that used the I/Cr ratio [81].

One study reported data for iodine sufficiency (as opposed to iodine deficiency) [32] and another for lowest vs. highest dairy intake (as opposed to highest vs. lowest dairy intake) [48]; we converted both to align with our outcome definition. Two studies reported results separately for different dairy types as well as total dairy; we used the data for total dairy in the meta-analysis. Three studies [48,49,77] reported data for separate dairy types, and we merged these within each study to generate an OR for total dairy intake. Two studies [34,65] reported data using different cut-offs for iodine deficiency (<150 μg/L and <50 μg/L, the latter representing severe deficiency); for consistency, we used the <150 μg/L definition, as it encompassed all other categories of iodine deficiency. Ferreira et al. [48] reported severe iodine deficiency data for supplement consumers, non-consumers, and for all participants combined. For the pooled analysis, we used the data for all participants combined.

Across the 11 independent cohorts, 8 [34,50,54,58,65,78,80,81] provided a single, non-overlapping exposure definition, totaling 1027 exposed and 1010 unexposed participants, with 638 deficiency events among the exposed. Three studies [48,49,77] reported overlapping dairy subtypes (milk, yogurt, and cheese); therefore, their exposed/unexposed counts were not combined to avoid double-counting. Another study did not report the number of participants in the exposed and unexposed groups [32]. In total, these 4 studies contributed a combined total of 3032 participants, each entered once into the meta-analysis using an averaged OR across the reported dairy subtypes.

**Figure 3:**
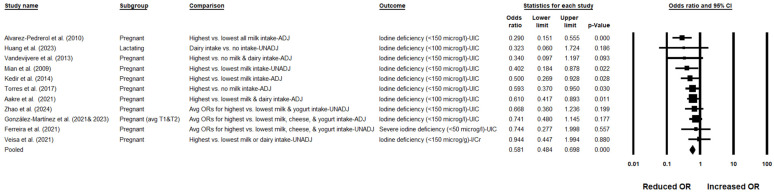
Meta-analysis of dairy intake and odds of iodine deficiency in pregnant and lactating women using a random-effects model (*n* = 12 publications) [32,34,48,49,50,54,58,65,77,78,80,81]. In this forest plot, each study or stratum is represented by a square indicating the point estimate, with horizontal lines showing the 95% CI. The square size reflects the relative weight of the study or stratum in the analysis. The diamond reflects the pooled estimate. ADJ = adjusted; Avg = average; CI = confidence interval; I/Cr = iodine-to-creatinine ratio; OR = odds ratio; T1 = first trimester; T2 = second trimester; UIC = urinary iodine concentration; UNADJ = unadjusted.

In the pooled analysis of 11 cohorts [32,34,48,49,50,54,58,65,77,78,80,81], higher dairy intake was associated with significantly lower odds of iodine deficiency (OR: 0.581; 95% CI: 0.484, 0.698; *p* < 0.001). Notably, there was no evidence of between-study heterogeneity (*I*^2^ = 0%) (Table 2 and Figure 3), and the certainty of evidence was found to be moderate (Supplementary Table S6). Importantly, these significant findings with low heterogeneity included the study by Ferreira et al. [48] which reported severe iodine deficiency, indicating that dairy intake was still associated with lower odds of iodine deficiency even when this study was included.

Visual inspection of the funnel plot indicated some asymmetry. The trim-and-fill method imputed 3 studies missing to the right of the pooled effect; the bias-adjusted pooled effect remained protective (OR: 0.631; 95% CI: 0.507, 0.784) (Supplementary Figure S7).

Because regions of China, including river basin areas, have naturally high iodine in soil and water, we excluded Zhao et al. [77] in a sensitivity analysis. The association between dairy intake and lower odds of iodine deficiency remained significant (OR: 0.569; 95% CI: 0.464, 0.699), with low heterogeneity (*I*^2^ = 7.794%), indicating the protective effect was not explained by populations living in iodine-rich regions.

We conducted a subgroup analysis based on adjustment status. Studies reporting adjusted estimates (6 studies [32,34,49,50,58,78,80]; OR: 0.559; 95% CI: 0.446, 0.701; *p* < 0.001; *I*^2^ = 23.267%) and those reporting unadjusted estimates (5 studies [48,54,65,77,81]; OR: 0.635; 95% CI: 0.436, 0.925; *p* = 0.018; *I*^2^ = 0%) both demonstrated a significant protective association of dairy intake with iodine deficiency, and the direction of effect was consistent across subgroups, with low heterogeneity observed in both (Table 2). To test robustness, we re-entered 6 studies with adjusted models using their unadjusted counts and combined them with 5 unadjusted studies (total studies = 11). The association remained significant, indicating a protective effect of dairy intake against iodine deficiency (OR: 0.655; 95% CI: 0.547, 0.784; *p* < 0.001; *I*^2^ = 19.921%).

Ferreira et al. [48] reported data for pregnant women who were non-supplement consumers and severely iodine deficient (UIC < 50 µg/L), as well as for those who were iodine deficient (UIC 50 to 100 µg/L). These groups were merged to derive an overall OR for iodine deficiency among supplement non-consumers. In addition, Alvarez-Pedrerol et al. [34] also provided OR data for iodine deficiency in non-supplement consumers. Pooled data showed higher dairy intake was associated with significantly lower odds of iodine deficiency among non-supplement consumers (OR: 0.273; 95% CI: 0.142, 0.525; *p* < 0.001; *I*^2^ = 0%) (Table 2).

Separate analyses restricted to studies that reported individual dairy products were conducted. Results indicated that the protective effect was most clearly attributable to milk intake (8 studies [34,48,49,50,58,65,77,78]; OR: 0.528; 95% CI: 0.408, 0.682; *p* < 0.001; *I*^2^ = 19.192%) and yogurt intake (4 studies [48,49,50,77]; OR: 0.707; 95% CI: 0.503, 0.995; *p* = 0.047; *I*^2^ = 4.654%), while the association for cheese (3 studies [48,49,50]; OR: 0.798; 95% CI: 0.514, 1.24; *p* = 0.316; *I*^2^ = 0%) was not significant (Table 2).

The 2 remaining studies, which did not provide data suitable for pooling, reported findings consistent with the overall trend. Farha et al. [47] observed lower milk consumption among women with UIC < 150 µg/L compared to those with UIC ≥ 150 µg/L. Similarly, Lean et al. [60] found higher milk product consumption in women with UIC in the highest quartile (sufficient range) at week 34 of pregnancy (27 vs. 10 times/month), while no difference was observed between UIC quartiles at week 17 (14 vs. 13 times/month).

In the study conducted among lactating women [54], iodine deficiency was also assessed using BMIC values. Event rates indicated fewer cases among higher dairy consumers compared to lower dairy consumers corresponding to an OR of 0.29 (95% CI: 0.05, 1.50). This suggests lower odds of iodine deficiency with higher dairy intake, although the effect estimate was associated with wide CIs and was not statistically significant.

##### Dietary Iodine Deficiency Based on Dairy Intake

Two studies [40,63] that assessed iodine deficiency using dietary intake data reported both adjusted and unadjusted estimates. Meta-analysis of the adjusted data (OR: 0.06; 95% CI: 0.01, 0.381; *p* = 0.003; *I*^2^ = 99.662%) and unadjusted data (OR: 0.081; 95% CI: 0.023, 0.287; *p* < 0.001; *I*^2^ = 99.334%) indicated statistically significant associations, with lower odds of iodine deficiency among women with higher dairy intake (Supplementary Table S7). Although the effect sizes were large, heterogeneity was extremely high, and the certainty of evidence was rated as moderate according to the GRADE assessment (Supplementary Table S6).

#### 3.4.3. Breast Milk Iodine Status Based on Dairy Intake

Six studies [57,66,71,72,75,79] reported BMIC in relation to dairy intake. Among these, 2 studies provided correlation coefficients [71,75] and 1 study reported geometric mean ratios [57]. The remaining 3 studies did not provide sufficient data to be pooled. Because the study reporting geometric mean ratios was not comparable with correlation coefficients, we conducted a random-effects meta-analysis using only the 2 studies with correlation coefficients to maintain homogeneity. The pooled correlation estimate was positive but not statistically significant (r = 0.247; 95% CI: −0.703, 1.98; *p* = 0.61), with substantial heterogeneity (*I*^2^ = 89.494%) (Supplementary Table S7).

Trabzuni et al. [79] reported that dairy intake had the most significant effect on BMIC in lactating women (*p* = 0.0003). Jorgensen et al. [57] reported a significant positive association between dairy intake and BMIC, supporting higher BMIC concentrations with greater dairy consumption. By contrast, Moon et al. [66] reported BMIC at 2 postpartum time points (2 to 5 days and 4 weeks) and found no statistically significant differences between the highest and lowest dairy consumers at either time period. Similarly, Prpić et al. [72] found no significant association between milk, yogurt, or cheese intake and BMIC in lactating women.

#### 3.4.4. Dietary Iodine Intake in Relation to Dairy Intake

Three studies [40,66,69] reported data on iodine intake from food in relation to dairy consumption. One study additionally provided data on total iodine intake (from both diet and supplements) by level of dairy consumption [79] and therefore is presented separately. Of these studies, only 2 provided sufficient data for pooling [40,69]. The pooled analysis based on dietary iodine intake showed substantially higher intake in the highest vs. lowest dairy consumption groups (SMD: 0.924; 95% CI: 0.794, 1.053; *p* < 0.001; *I*^2^ = 4.936%) (Supplementary Table S7), and the overall certainty of evidence was rated as moderate (Supplementary Table S6).

In their large cohort study, Brantsaeter et al. [40] reported that the highest dairy consumers had a significantly higher total iodine intake (from both diet and supplements) (mean [SD]: 249.3 [208.3] μg/day) compared with the lowest dairy consumers (mean [SD]: 139.0 [154.9] μg/day). In contrast, Moon et al. [66] reported no significant differences in dietary iodine intake between the highest and lowest dairy intake groups at 2 postpartum time points (2 to 5 days and 4 weeks).

#### 3.4.5. The Contribution of Dairy Iodine Intake to Overall Iodine Intake

Eleven studies [35,39,40,43,44,52,69,74,76,79,82] reported the percentage contribution of iodine intake from dairy to overall iodine intake. Of these, nine studies reported the contribution of dairy to iodine intake from foods only [35,39,43,44,52,69,74,76,79], one study reported the contribution of dairy to total iodine intake (defined as iodine from foods plus supplements) [40], and one study reported the contribution of dairy to both dietary iodine intake and total iodine intake separately [82]. Among the nine studies reporting the contribution of dairy-derived iodine to iodine intake from foods, it should be noted that each used slightly different definitions of total dietary iodine, with some including iodized salt [43,69,74], some including water [43,74], and others providing very little detail. Two studies [39,52] provided data using both FFQ and other dietary assessment methods (e.g., 24-h recall [52] or food diary [39]); for consistency, we used the FFQ data. In the study by Charlton et al. [43], datasets were available for 2 time periods (2011 and 2012); we included only the 2011 data, as the sample size was larger and to avoid overlap with the 2012 dataset. We therefore calculated the weighted mean percentage contribution of dairy iodine intake to iodine intake from foods and to total iodine intake. Based on 10 studies, the contribution of dairy iodine intake to iodine intake from foods was 27%. Based on 2 studies, the contribution of dairy iodine intake to total iodine intake was 51%.

#### 3.4.6. The Intervention Study

The RCT study [67] was conducted in 84 healthy lactating mother-infant pairs from health care centers in Iran. Mothers (age [mean ± SD]: 28.2 ± 4.5 years) were randomly assigned to consume either iodine-fortified milk (approximately 150 µg/day in 200 mL of milk) (*n* = 42 women) or no intervention (*n* = 42 women). Both groups were instructed to consume iodized salt during cooking and as table salt. Supplementation started on the sixth day postpartum and continued for 4 weeks. Urine and milk samples were collected from each lactating mother at baseline and 7 days, 10 days, 14 days, and 1 month postpartum. Compared with lactating women in the control group, those who received iodine-fortified milk had significantly higher UIC and BMIC at the end of the study (*p* < 0.001).

## 4. Discussion

In this systematic review and meta-analysis, we synthesized evidence from 51 studies to provide a comprehensive overview of the association between dairy intake and iodine status in pregnant and lactating women. We found that higher dairy intake was significantly associated with improved urinary iodine status and reduced odds of iodine deficiency, with consistent findings across SMD, beta coefficient/correlation, and OR estimates. Subgroup analyses further demonstrated stronger associations in specific settings (e.g., Eastern Europe, women in the third trimester or in summer season, studies of higher methodological quality, and populations consuming mainly milk as the dairy type), and among both supplement consumers and non-consumers, indicating that the positive association of dairy intake with iodine status was robust across populations. BMIC results were less consistent, with limited pooled data showing no significant associations, although some individual studies suggested protective effects. Dietary iodine intake was substantially higher among high vs. low dairy consumers, and dairy contributed, on average, 27% of dietary iodine intake. Finally, an RCT in lactating women confirmed that iodine-fortified milk improved both UIC and BMIC compared with controls.

The daily iodine requirement during pregnancy is increased by ~50% to ensure sufficient placental transfer of iodine to the fetal thyroid for adequate hormone production [2]. The WHO defines adequate iodine status in pregnancy and lactation as a median UIC of 150 to 249 μg/L [83]. There is a resurgence of iodine deficiency in pregnant and lactating women in economically developed countries. In a meta-analysis of observational studies, the global prevalence of insufficient iodine intake in pregnant women was 53%, and remained high at 51%, even in countries considered iodine-adequate or iodine-replete [12]. In Brazil, the median UIC of 273 pregnant women was 146 μg/L, with 52% classified as iodine deficient, despite residing in an area reported to have adequate iodine status [84]. In the United States, the prevalence of inadequate iodine status among pregnant women ranged from 23 to 59%, with dietary changes, particularly reduced milk consumption, identified as the main contributor [85]. Similarly, Zimmermann et al. [86] noted that two-thirds of European countries assessing iodine nutrition during pregnancy reported inadequate iodine intake. For example, median UIC values were well below adequacy thresholds in Sweden (98 μg/L; *n* = 459) [87] and Austria (87 μg/L; *n* = 246) [88]. In New Zealand, iodine deficiency was even more pronounced, with a median UIC of only 38 μg/L and a mean iodine intake of 48 μg/day among 170 pregnant women [89]. Further supporting these observations, a recent longitudinal analysis from Australia found that women consumed less dairy and had lower iodine intake at 1-year postpartum compared with late pregnancy, alongside persistently suboptimal diet quality [90]. These findings reinforce that declining consumption of iodine-rich foods contributes to inadequate iodine status during pregnancy and lactation and highlight the need to improve awareness of iodine-rich dietary sources among pregnant and postpartum women.

Several factors likely contribute to the resurgence of iodine deficiency in pregnant and lactating women. One important contributor is the avoidance of iodized salt during pregnancy and lactation, often motivated by concerns about edema, gestational hypertension, or preeclampsia [91]. Increased sodium intake has been associated with an increased risk of hypertensive disorders of pregnancy [19,92]. Indeed, several recommendations for pregnant and lactating women encourage reduced salt intake. For instance, according to Health Canada’s Food Guide, pregnant and lactating women should “*choose foods that have little to no added sodium, sugars or saturated fat*” [93]. Likewise, according to the Nordic Nutrition Recommendations, pregnant women should limit their intakes of salt [94]. Observational studies demonstrate that iodine status in pregnancy is closely tied to iodized salt intake, with stepwise increases in median UIC observed as salt iodine content rises [95]. However, iodized salt alone may not provide sufficient iodine to pregnant women when overall salt intake is reduced [91]. Taken together, these findings indicate that although iodized salt is an important contributor to maternal iodine intake, intentional avoidance or restricted salt intake during pregnancy and lactation may compromise iodine sufficiency, with implications for both maternal thyroid function and fetal development.

A second factor that may contribute to the resurgence of iodine deficiency in pregnant and lactating women is the avoidance or low intake of seafood and fish, which represent important dietary iodine sources. In high-income countries, observational data consistently show that lower seafood consumption is associated with lower dietary iodine intake and UIC in pregnant and lactating women [51]. Despite fish providing nearly half of iodine intake in some countries (e.g., Iceland and Norway), actual consumption among pregnant and lactating women remains well below national recommendations [17,33,51]. In other countries, fish contributes <15% of total iodine intake, which may further exacerbate iodine insufficiency in pregnant and lactating women. Multiple influences likely contribute to this avoidance, including socioeconomic constraints, concerns about heavy metal exposure, gastrointestinal tolerance, and perceived or actual allergy risk. In the United States, pregnant and postpartum women frequently limit seafood intake due to concerns about fetal harm or infant allergy risk, despite Food and Drug Administration recommendations to consume 8 to 12 ounces of low-mercury seafood weekly [96]. Together, these findings highlight socioeconomic, safety, and informational barriers to seafood consumption as potential drivers of inadequate iodine intake.

A third factor that may contribute to the resurgence in iodine deficiency in pregnant and lactating women relates to the global shift from animal-based to plant-based dietary patterns, including increased consumption of plant-based alternatives [97]. While dairy products remain one of the richest sources of iodine in developed countries, plant-based alternatives generally contain very low iodine concentrations and are rarely fortified. Consequently, individuals who follow vegan or vegetarian diets, or who frequently consume plant-based substitutes for milk and dairy are at elevated risk of iodine insufficiency [97,98], particularly in countries without mandatory fortification or universal salt-iodization programs, such as Norway or the United Kingdom [76]. Several studies confirm this concern: vegans and vegetarians report lower iodine intakes compared with omnivores [99,100], and iodine supplementation is not commonly practiced in these groups. Importantly, lactating women consuming vegetarian or vegan diets have been shown to have significantly lower BMIC than omnivores in Spain and the United States [101,102]. Similar findings in the United Kingdom suggest that mothers with greater intake of plant-based milk alternatives are less likely to meet recommended iodine intakes [103]. These observations highlight that although plant-based diets may offer health and environmental benefits, without iodine fortification or “supplementation” they may inadvertently exacerbate iodine deficiency during pregnancy and lactation.

Our findings that higher dairy intake was significantly associated with improved iodine status, higher dietary iodine intake, and lower odds of iodine deficiency in pregnant and lactating women can be explained by several mechanisms. Dairy products represent one of the richest and most reliable dietary sources of iodine in many countries, owing predominantly to iodized cattle feed as well as additional contributions from the treatment of lactating udders with iodine pre-milking [104,105]. A randomized crossover study in 12 adults found that iodine from cow’s milk (either intrinsic to the milk or extrinsically added to the milk) is highly bioavailable (~91%), with 80 to 93% of milk iodine present as inorganic iodide, confirming milk as an efficient and reliable contributor to iodine nutrition [106]. Although, in this same study, iodine from an aqueous iodine solution was found to have similar bioavailability as iodine from milk, milk has very high quality protein as well as other nutritional attributes which make it an optimal food for pregnant and lactating women (discussed further below).

Based on our results, analyses restricted to milk as the primary dairy type revealed stronger inverse associations, such that greater milk intake was associated with better iodine status and reduced odds of iodine insufficiency. Consistent with our findings, a 12-week RCT in 78 healthy women demonstrated that increasing cow’s milk consumption significantly improved UIC at weeks 6 and 12 compared with controls, indicating that milk can meaningfully improve iodine status in women of childbearing age [107]. This supports the role of milk as an important dietary iodine source, particularly as iodine concentrations are higher in cow’s milk than in other dairy products [108], and milk is the most frequently consumed dairy type [109].

We also observed stronger associations in studies from Eastern Europe. A recent systematic review showed that baseline iodine intake in Europe is often below recommendations, with the greater inadequacy in Eastern European countries and particularly among women of reproductive age [17]. These differences appear to be driven by inconsistent iodization policies and lower consumption of iodine-rich foods, including dairy, iodized salt, and “supplements” during life stages with higher requirements. Collectively, this highlights the need for region-specific strategies to address iodine insufficiency, particularly in vulnerable groups.

In subgroup analyses, we also found stronger associations in women studied during the third trimester of pregnancy. This is biologically plausible, as iodine requirements increase progressively across pregnancy due to higher maternal thyroid hormone production, fetal thyroid development, and enhanced renal clearance of iodine [15,110]. Women with greater dairy intake are therefore better positioned to meet these elevated demands, and the benefits of dairy consumption become particularly evident in late gestation, when the risk of deficiency is highest.

Interestingly, heterogeneity was markedly lower among studies conducted in the summer. This likely reflects the fact that season influences both iodine concentration in milk (through cattle feed composition and pasture access) [111] and UIC [112]. A systematic review of 66 data sources from 34 countries similarly showed that milk-iodine concentration varies widely (5.5 to 49.9 μg/100 g; median 17.3 μg/100 g), with significantly higher levels in winter than summer milk [113]. These seasonal variations underscore the importance of accounting for milk iodine variability when interpreting associations with iodine status. This explanation is also consistent with findings from Bath et al. [36], who reported a significant interaction between milk intake and season, showing that the association between dairy intake and iodine status was stronger in winter compared with summer. A further possible explanation is that the lower heterogeneity we observed may simply reflect a methodological artifact, since relatively few studies explicitly evaluated season as a modifier or conducted subgroup analyses by season. Additional research is therefore needed to confirm whether seasonal variation truly explains the reduced heterogeneity observed in summer-only studies.

We also observed that the association between dairy intake and iodine status was consistent in both supplement consumers and non-consumers, underscoring the robustness of the relationship. The fact that the association remained significant among non-consumers confirms that the findings are not driven solely by supplement use. For supplement consumers, it is possible that inconsistent adherence reduced the expected benefit of supplementation, making habitual dairy intake an important complementary source of iodine. Together, these findings suggest that dairy contributes meaningfully to iodine status across different intake patterns and supplementation behaviors.

The present meta-analysis showed that higher methodological quality strengthened the associations. Studies that applied validated dietary assessment tools, used standardized urinary iodine assays, and carefully adjusted for key confounders such as supplement use and iodized salt intake yielded more consistent results with reduced heterogeneity. These design features reduce measurement error and residual confounding, thereby yielding more precise estimates of the association between dairy intake and iodine status.

On the other hand, iodine intake and thyroid status remain important considerations even in developed countries generally regarded as iodine-sufficient. For example, 2 included studies (1 in the United Kingdom [76] and 1 in Belgium [80]) reported goiter among pregnant women, an unexpected finding in these settings. This underscores the importance of dairy as a reliable iodine source for women, including those in countries assumed to have adequate iodine intake.

Some limitations of our systematic review and meta-analysis should be acknowledged. Most studies included in our review were conducted in European countries, which may limit the generalizability of our findings to regions with different dietary patterns or cattle feed iodine fortification policies. The certainty of evidence for most outcomes was generally low to very low, with only a few outcomes rated as moderate, thereby reflecting the cross-sectional design of most of the included studies and underscoring the need for well-designed RCTs to draw stronger conclusions. Additionally, there were important differences across the studies, including the tool used to assess dietary intakes and its validity specifically in pregnant or lactating women; the analytical method used to measure urinary iodine (e.g., Sandell–Kolthoff or HPLC); the differential adjustments for relevant covariates (e.g., supplement/iodized salt use); and the variability in the objective investigation of thyroid disorders. Each of these factors may have influenced iodine status in the participants and contributed to heterogeneity.

Our study has several notable strengths. This is the first systematic review and meta-analysis to specifically investigate the association between dairy intake—the major dietary source of iodine in many populations—and iodine status during pregnancy and lactation, periods in which adequate iodine intake is critical for maternal and infant health. We conducted a comprehensive and systematic search of the literature using a broad range of keywords to minimize the likelihood of missing relevant studies. The inclusion of 51 publications representing diverse populations provided strong statistical power, and the consistent associations observed across multiple outcome measures (urinary and dietary iodine status, iodine deficiency, dietary iodine intake, and RCT evidence) further strengthen confidence in the findings. In addition, the use of PRISMA guidelines and GRADE assessments enhanced methodological rigor and transparency. We performed multiple subgroup and sensitivity analyses to ensure the robustness of our findings. These included evaluating different urinary iodine measures (UIC and I/Cr) in the main meta-analysis, examining study quality, trimester, season, supplement use, confounder adjustment status, and conducting analyses restricted to specific dairy types.

Milk is an affordable, nutritionally dense beverage that delivers multiple essential nutrients with a modest caloric load [114]. Beyond being a leading source of iodine, milk contains casein (one of the highest quality proteins), as well as vitamin B12, riboflavin, choline, and several other nutrients (phosphorus, potassium, magnesium, zinc, and selenium), all of which are important during pregnancy and lactation when requirements are generally increased [115]. With a water content of approximately 87%, milk is also a hydrating beverage [116]. The intake of milk during pregnancy supports not only iodine sufficiency but also overall nutritional adequacy in these life stages.

## 5. Conclusions

Higher dairy intake was associated with better urinary iodine status and lower odds of iodine deficiency, with consistent signals across SMDs, beta coefficient/correlation, and OR analyses. Notably, most evidence came from pregnant cohorts. Effects were stronger when milk was the primary dairy type, in Eastern European populations, and in late pregnancy, consistent with higher physiological requirements. Heterogeneity was lower in higher-quality studies. Evidence for BMIC was limited, yielding a non-significant pooled effect, and showed substantial between-study heterogeneity.

To clarify effects and reduce heterogeneity, future systematic reviews and meta-analyses should: (i) standardize exposure definitions (dairy type, portion size, milk vs. yogurt/cheese; production system; and season) and outcome measures (spot UIC with creatinine, 24-h UIE, and BMIC); (ii) adjust in detail for iodine-containing foods and supplements; (iii) stratify by trimester and baseline iodine sufficiency; (iv) ascertain thyroid disorder status using laboratory criteria (exclude or analyze separately); (v) incorporate dose–response and region-specific analyses; and (vi) use detailed, validated dietary questionnaires tailored to pregnant and lactating women, with documented reproducibility and validation against biomarkers (UIC and BMIC). Primary studies should prioritize prospective designs and RCTs, harmonize BMIC protocols, and pre-register protocols, especially in lactating women, where evidence remains sparse.

Insufficient iodine intake during pregnancy remains a concern not only in low-resource settings but also in high-income countries such as the United Kingdom and Belgium. These findings underscore the need for strengthened public health strategies, including clearer dietary guidance and reinforced education on iodine-rich foods for pregnant and postpartum women. Continued support for effective iodization programs and enhanced global advocacy, such as through World Health Organization initiatives, will be essential to safeguard maternal and fetal iodine sufficiency.

## Figures and Tables

**Table 1:** The association between dairy intake and urinary iodine status: meta-analysis results ^a^.

Meta-Analysis				Heterogeneity		
Study Group	Number of Studies	Effect Estimate (95% CI) ^b^	*p* Effect	Q Statistic	*p* Heterogeneity	*I*^2^ (%)
Overall	23	0.326 (0.228, 0.424)	<0.001	51.538	<0.001	57.313
Country—Geographically based						
Eastern European	2	0.435 (0.05, 0.82)	0.027	0.181	0.67	0.0
Western European	16	0.382 (0.277, 0.488)	<0.001	36.19	0.002	58.552
Others	5	0.084 (−0.116, 0.284)	0.41	4.126	0.389	3.057
Quality of studies ^c^						
High	3	0.525 (0.265, 0.785)	<0.001	1.654	0.437	0.0
Medium	17	0.254 (0.153, 0.354)	<0.001	31.369	0.012	48.995
Low	3	0.576 (0.32, 0.832)	<0.001	4.627	0.099	56.771
Pregnancy trimester						
First	12	0.336 (0.192, 0.480)	<0.001	30.218	0.001	63.579
Second	3	0.172 (−0.092, 0.436)	0.202	4.599	0.1	56.513
Third	6	0.390 (0.196, 0.585)	<0.001	7.702	0.173	35.08
All	2	0.336 (−0.001, 0.673)	0.05	3.049	0.081	67.202
Seasonality						
Summer	4	0.299 (0.022, 0.576)	0.034	1.811	0.613	0.0
Winter-to-summer	5	0.337 (0.116, 0.558)	0.003	20.012	<0.001	80.012
All seasons	7	0.388 (0.21, 0.566)	<0.001	15.891	0.014	62.242
Not reported	7	0.262 (0.069, 0.455)	0.008	10.649	0.1	43.656
Use of iodine (or iodine containing) supplements						
Non-consumers	2	0.396 (0.05, 0.742)	0.025	0.551	0.458	0.0
Consumers	2	0.508 (0.027, 0.988)	0.039	1.527	0.217	34.493
Type of dairy (overall dairy vs. milk)						
Total dairy	17	0.298 (0.183, 0.413)	<0.001	43.976	<0.001	63.616
Milk	6 ^d^	0.405 (0.212, 0.598)	<0.001	5.071	0.407	1.393
Type of dairy (sensitivity analysis by dairy type) ^e^						
Milk	13 ^f^	0.420 (0.268, 0.571)	<0.001	32.838	0.001	63.457
Cheese	3	0.167 (0.013, 0.32)	0.034	2.089	0.352	4.263
Yogurt	3	0.444 (−0.406, 1.295)	0.306	35.974	<0.001	94.44

CI = confidence interval. ^a^ The main analysis was conducted using a random-effects model. Subgroup analyses were conducted using a mixed-effects model. ^b^ The effect estimate represents the pooled standardized mean difference. ^c^ Study quality was assessed using the Observational Study Quality Evaluation tool and categorized as low, medium, or high according to total scores. ^d^ For these 6 studies, only intakes of milk were reported; consequently, milk intakes were used as a proxy for overall dairy intake in the main analysis. ^e^ Individual random-effects analyses were performed for each dairy type (milk, cheese, and yogurt). ^f^ These 13 studies include the 6 in which only milk intakes were reported, as well as an additional 7 studies in which milk intakes were reported separately, along with the separate reporting of other dairy intakes (e.g., overall dairy, yogurt, and cheese).

**Table 2:** The association between dairy intake and risk of iodine deficiency: meta-analysis results ^a^.

Meta-Analysis				Heterogeneity		
Study Group	Number of Studies	Odds Ratio (95% CI)	*p* Effect	Q Statistic	*p* Heterogeneity	*I*^2^ (%)
Overall	11	0.581 (0.484, 0.698)	<0.001	9.973	0.443	0.0
Adjustment status						
Adjusted (most adjusted model reported)	6	0.559 (0.446, 0.701)	<0.001	6.516	0.259	23.267
Unadjusted (crude estimates)	5	0.635 (0.436, 0.925)	0.018	3.146	0.534	0.0
Use of iodine (or iodine containing) supplements ^b^						
Non-consumers	2	0.273 (0.142, 0.525)	<0.001	0.517	0.472	0.0
Type of dairy ^b^						
Milk	8	0.528 (0.408, 0.682)	<0.001	8.663	0.278	19.192
Cheese	3	0.798 (0.514, 1.24)	0.316	0.063	0.969	0.0
Yogurt	4	0.707 (0.503, 0.995)	0.047	3.146	0.37	4.654

CI = confidence interval. ^a^ Main analysis was conducted using a random-effects model. Subgroup analyses were conducted using a mixed-effects model. ^b^ Individual random-effects analyses were performed for each dairy type.

## Data Availability

The raw data supporting the conclusions of this article will be made available by the authors on request. The listing of excluded studies can also be provided upon request.

## Supplementary Materials

The following supporting information can be downloaded at: https://www.mdpi.com/article/10.3390/nu17233765/s1, Supplementary Figure S1: Sensitivity (leave-one-out) analysis for dairy intake and urinary iodine status in pregnant women (studies in lactating women not identified) (*n* = 23 publications) [33,34,36,37,38,41,45,48,50,51,53,55,56,58,59,62,64,65,68,69,70,78,81]. If a study reported UIC as well as other measures of urinary iodine, UIC was used in the meta-analysis, as it was the most commonly reported outcome across studies. Each square and horizontal line within each row show the overall pooled estimate and 95% CI, respectively, with the study in the corresponding row left out; Supplementary Figure S2: Funnel plot of standard error by Std diff in means for dairy intake and urinary iodine status using a random-effects model in pregnant women (studies in lactating women not identified) (*n* = 23 publications) [33,34,36,37,38,41,45,48,50,51,53,55,56,58,59,62,64,65,68,69,70,78,81]. If a study reported UIC as well as other measures of urinary iodine, UIC was used in the meta-analysis, as it was the most commonly reported outcome across studies. Publication bias was not detected, and the trim-and-fill method did not impute any missing studies; Supplementary Figure S3: Meta-analysis of dairy intake and urinary iodine status using a random effects model in pregnant women (studies in lactating women not identified)—sensitivity analysis based on method of urinary iodine status assessment (*n* = 23 publications) [33,34,36,37,38,41,45,48,50,51,53,55,56,58,59,62,64,65,68,69,70,78,81]. In this forest plot, each study or stratum is represented by a square indicating the point estimate, with horizontal lines showing the 95% confidence interval. The square size reflects the relative weight of the study or stratum in the analysis. The diamond reflects the pooled estimate. In this meta-analysis, for the five studies in which urinary iodine status was assessed using both UIC and I/Cr, I/Cr data were used instead of UIC as a sensitivity analysis. The association remained statistically significant such that urinary iodine status was significantly greater with higher dairy intake (SMD = 0.355; 95% CI: 0.236, 0.474; *p* < 0.001; *I*^2^ = 71.38%); Supplementary Figure S4: Meta-analysis of dairy intake and urinary iodine status in pregnant and lactating women using a random-effects model (Fisher’s Z converted from beta and correlation coefficients) (*n* = 8 publications) [39,45,46,55,56,70,73,75]. In this forest plot, each study or stratum is represented by a square indicating the point estimate, with horizontal lines showing the 95% confidence interval. The square size reflects the relative weight of the study or stratum in the analysis. The diamond reflects the pooled estimate. Pooling the results yielded a significantly greater urinary iodine status with higher dairy intake (SMD: 0.228; 95% CI: 0.154, 0.301; *p* < 0.001; *I*^2^ = 59.495%); Supplementary Figure S5: Funnel plot of standard error by Std diff in means for dairy intake and urinary iodine status using a random-effects model in pregnant and lactating women (Fisher’s Z converted from *beta* and correlation coefficients) (*n* = 8 publications) [39,45,46,55,56,70,73,75]. The trim-and-fill method imputed 1 study missing to the left of the pooled effect; with this study imputed, the SMD remained significant such that urinary iodine status was significantly greater with higher dairy intake (SMD: 0.226; 95% CI: 0.143, 0.292); Supplementary Figure S6: Sensitivity (leave-one-out) analysis for dairy intake and urinary iodine status in pregnant and lactating women (Fisher’s Z converted from beta and correlation coefficients) (*n* = 8 publications) [39,45,46,55,56,70,73,75]. Each square and horizontal line within each row show the overall pooled estimate and 95% CI, respectively, with the study in the corresponding row left out; Supplementary Figure S7: Funnel plot of standard error by log OR for dairy intake and odds of iodine deficiency using a random-effects model in pregnant and lactating women (*n* = 12 publications) [32,34,48,49,50,54,58,65,77,78,80,81]. The trim-and-fill method imputed 3 studies missing to the right of the pooled effect; the bias-adjusted pooled effect remained protective such that there was a significant reduction in the odds of iodine deficiency with higher dairy intake (OR: 0.631; 95% CI: 0.507, 0.784); Supplementary Table S1: Electronic databases used to retrieve literature; Supplementary Table S2: Keywords used to retrieve literature; Supplementary Table S3: Key characteristics of studies conducted in pregnant women (*n* = 42 publications, 42 studies); Supplementary Table S4: Key characteristics of studies conducted in lactating women (*n* = 9 publications, 9 studies); Supplementary Table S5: Summary of study quality appraisals (*n* = 51); Supplementary Table S6: GRADEPro assessment of iodine status outcomes in relation to dairy intake during pregnancy and lactation; Supplementary Table S7: Meta-analysis showing the association between dairy intake and different iodine status outcomes.

## Abbreviations

The following abbreviations are used in this manuscript:

ALSPAC	Avon Longitudinal Study of Parents and Children
BMIC	breast milk iodine concentration
CI	confidence interval
CMA	Comprehensive Meta-Analysis
FFQ	food frequency questionnaire
GRADE	Grading of Recommendations, Assessment, Development, and Evaluations
HPLC	high-performance liquid chromatography
I/Cr	iodine-to-creatinine ratio
INMA	INfancia y Medio Ambiente
IQR	interquartile range
MD	mean difference
NOS	Newcastle-Ottawa Scale
OR	odds ratio
OSQE	Observational Study Quality Evaluation
PRISMA	Preferred Reporting Items for Systematic Reviews and Meta-Analyses
PROSPERO	International Prospective Register of Systematic Reviews
RCT	randomized controlled trial
ROB2	Risk of Bias 2
SD	standard deviation
SMD	standardized mean difference
UIC	urinary iodine concentration
UIE	urinary iodine excretion
USI	universal salt iodization
WHO	World Health Organization
