# Supplementary material for: Dairy Intake and Iodine Status in Pregnant and Lactating Women: A Systematic Review and Meta-Analysis
Source: Nutrients. 2025 Nov 30;17(23):3765. doi: 10.3390/nu17233765 (PMC12693841; doi:10.3390/nu17233765)
Supplement: Supplementary file 1 [file nutrients-17-03765-s001.zip › Fig S1_Sensitivity_Dairy & Overall Urine Iodine_UIC_SMD_ 25Nov2025.pdf]

Study name	Subgroup	Comparison	Outcome	Statistics with study removed				
				Point	Standard error	Lower limit	Upper limit	p-Value
Huang et al. (2020)	Pregnant	Highest vs. lowest dairy intake	Iodine status (UIC)	0.343	0.049	0.247	0.439	0.000
Blumenthal et al. (2012)	Pregnant	Avg SMD for highest vs. lowest milk & dairy intake	Iodine status (UIC)	0.342	0.049	0.245	0.439	0.000
González-Martínez et al. (2021)	Pregnant	Highest vs. lowest dairy intake	Iodine status (UIC)	0.340	0.051	0.240	0.440	0.000
Ollero et al. (2020)	Pregnant	Highest vs. lowest dairy intake	Iodine status (UIC)	0.338	0.052	0.237	0.439	0.000
Johannesen et al. (2024)	Pregnant	Avg SMD for cheese & milk intake vs. no intake	Iodine status (UIC)	0.336	0.053	0.233	0.440	0.000
Ferreira et al. (2021)	Pregnant	Avg SMD for highest vs. lowest milk, cheese, & yogurt intake	Iodine status (UIC)	0.336	0.051	0.236	0.436	0.000
Perrine et al. (2010)	Pregnant	Dairy intake vs. no intake	Iodine status (UIC)	0.335	0.052	0.234	0.436	0.000
Kedir et al. (2014)	Pregnant	Highest vs. lowest milk intake	Iodine status (UIC)	0.334	0.053	0.230	0.438	0.000
Knight et al. (2017)	Pregnant	Avg SMD for highest cheese, milk & yogurt intake vs. no intake	Iodine status (UIC)	0.331	0.051	0.231	0.431	0.000
Menéndez Torre et al. (2014)	Pregnant	Highest vs. lowest dairy intake	Iodine status (UIC)	0.330	0.052	0.228	0.432	0.000
Opazo et al. (2023)	Pregnant	Highest vs. lowest milk intake	Iodine status (UIC)	0.326	0.051	0.227	0.426	0.000
Cannas et al. (2023)	Pregnant	Avg SMD for highest vs. lowest milk & dairy intake	Iodine status (I/Cr)	0.326	0.051	0.226	0.426	0.000
Adalsteinsdottir et al. (2020)	Pregnant	Highest vs. lowest dairy intake	Iodine status (UIC)	0.321	0.052	0.219	0.424	0.000
Johnsen et al. (2025)	Pregnant	Dairy intake vs. no intake	Iodine status (UIC)	0.321	0.051	0.220	0.422	0.000
Bath et al. (2014)	Pregnant	Highest vs. lowest milk intake	Iodine status (24-h UIE)	0.320	0.050	0.222	0.418	0.000
Veisa et al. (2021)	Pregnant	Highest vs. lowest dairy intake	Iodine status (I/Cr)	0.320	0.051	0.219	0.420	0.000
Torres et al. (2017)	Pregnant	Highest vs. no milk intake	Iodine status (UIC)	0.319	0.052	0.216	0.421	0.000
Mian et al. (2009)	Pregnant	Highest vs. lowest milk intake	Iodine status (UIC)	0.318	0.052	0.216	0.420	0.000
Alvarez-Pedrerol et al. (2010)	Pregnant	Highest vs. lowest all milk intake	Iodine status (UIC)	0.318	0.052	0.216	0.420	0.000
Gunnarsdottir et al. (2013)	Pregnant	Highest vs. lowest dairy intake	Iodine status (UIC)	0.316	0.051	0.216	0.416	0.000
Dahl et al. (2018)	Pregnant	Highest vs. lowest dairy intake	Iodine status (UIC)	0.309	0.050	0.211	0.407	0.000
Bath et al. (2015)	Pregnant	Avg SMD for highest vs. lowest milk & dairy intake	Iodine status (I/Cr)	0.308	0.049	0.212	0.405	0.000
McMullan et al. (2019)	Pregnant	Avg SMD for highest vs. lowest milk & full-fat yogurt intake	Iodine status (I/Cr)	0.306	0.047	0.214	0.397	0.000
Pooled				0.326	0.050	0.228	0.424	0.000

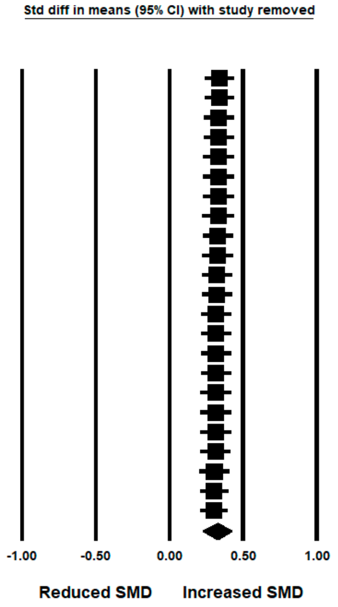

Supplementary Figure S1: Sensitivity (leave-one-out) analysis for dairy intake and urinary iodine status in pregnant women (studies in lactating women not identified) (*n* = 23 publications) [33,34,36–38,41,45,48,50,51,53,55,56,58,59,62,64,65,68–70,78,81]. If a study reported UIC as well as other measures of urinary iodine, UIC was used in the meta-analysis, as it was the most commonly reported outcome across studies. Each square and horizontal line within each row show the overall pooled estimate and 95% CI, respectively, with the study in the corresponding row left out. Avg = average; CI = confidence interval; h = hour; I/Cr = iodine-to-creatinine ratio; SMD = standardized mean difference; Std diff = standardized difference; UIC = urinary iodine concentration; UIE = urinary iodine excretion.
