# Supplementary material for: Dairy Intake and Iodine Status in Pregnant and Lactating Women: A Systematic Review and Meta-Analysis
Source: Nutrients. 2025 Nov 30;17(23):3765. doi: 10.3390/nu17233765 (PMC12693841; doi:10.3390/nu17233765)
Supplement: Supplementary file 1 [file nutrients-17-03765-s001.zip › Fig S2_PB_Dairy & Overall Urine Iodine_UIC_SMD_ 25Nov2025.pdf]

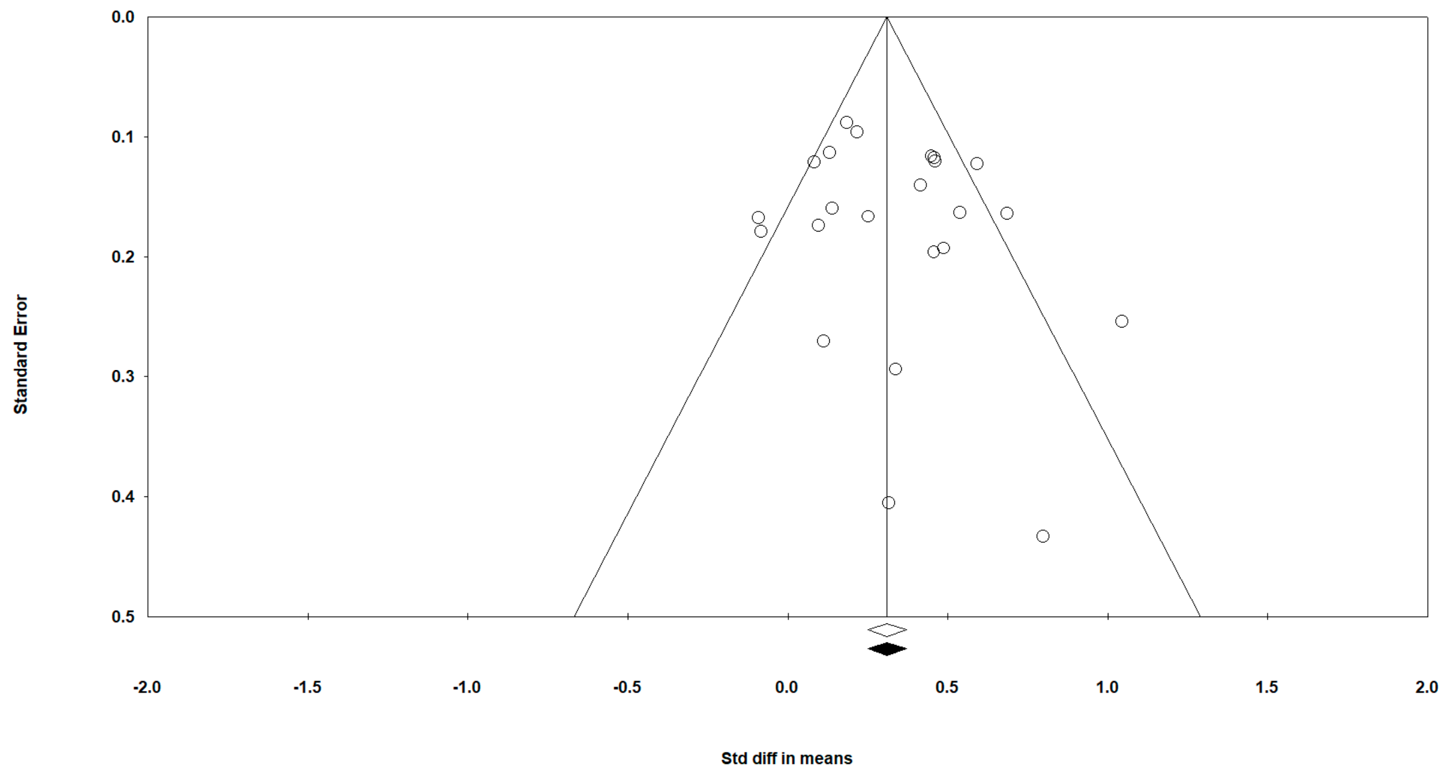

Supplementary Figure S2: Funnel plot of standard error by Std diff in means for dairy intake and urinary iodine status using a random-effects model in pregnant women (studies in lactating women not identified) (*n* = 23 publications) [33,34,36–38,41,45,48,50,51,53,55,56,58,59,62,64,65,68–70,78,81]. If a study reported UIC as well as other measures of urinary iodine, UIC was used in the meta-analysis, as it was the most commonly reported outcome across studies. Publication bias was not detected, and the trim-and-fill method did not impute any missing studies. Std diff = standardized difference; UIC = urinary iodine concentration.
