# Supplementary material for: Dairy Intake and Iodine Status in Pregnant and Lactating Women: A Systematic Review and Meta-Analysis
Source: Nutrients. 2025 Nov 30;17(23):3765. doi: 10.3390/nu17233765 (PMC12693841; doi:10.3390/nu17233765)
Supplement: Supplementary file 1 [file nutrients-17-03765-s001.zip › Fig S3_Dairy & Overall Urine Iodine_ICR_SMD_ 25Nov2025.pdf]

Study name	Subgroup	Comparison	Outcome	Std diff in means	Standard error	Lower limit	Upper limit	p-Value
McMullan et al. (2019)	Pregnant	Avg SMD for highest vs. lowest milk & full-fat yogurt intake	Iodine status (I/Cr)	1.045	0.254	0.547	1.543	0.000
Adalsteinsdottir et al. (2020)	Pregnant	Highest vs. lowest dairy intake	Iodine status (I/Cr)	1.010	0.146	0.724	1.296	0.000
Bath et al. (2014)	Pregnant	Highest vs. lowest milk intake	Iodine status (24-h UIE)	0.798	0.433	-0.051	1.647	0.065
Dahl et al. (2018)	Pregnant	Highest vs. lowest dairy intake	Iodine status (I/Cr)	0.731	0.124	0.489	0.974	0.000
Bath et al. (2015)	Pregnant	Avg SMD for highest vs. lowest milk & dairy intake	Iodine status (I/Cr)	0.686	0.164	0.364	1.007	0.000
Veisa et al. (2021)	Pregnant	Highest vs. lowest dairy intake	Iodine status (I/Cr)	0.488	0.193	0.110	0.866	0.011
Alvarez-Pedrerol et al. (2010)	Pregnant	Highest vs. lowest all milk intake	Iodine status (UIC)	0.461	0.120	0.225	0.697	0.000
Mian et al. (2009)	Pregnant	Highest vs. lowest milk intake	Iodine status (UIC)	0.457	0.117	0.227	0.688	0.000
Johnsen et al. (2025)	Pregnant	Dairy intake vs. no intake	Iodine status (UIC)	0.457	0.196	0.073	0.841	0.020
Torres et al. (2017)	Pregnant	Highest vs. no milk intake	Iodine status (UIC)	0.449	0.116	0.222	0.676	0.000
Cannas et al. (2023)	Pregnant	Avg SMD for highest vs. lowest milk & dairy intake	Iodine status (I/Cr)	0.339	0.294	-0.237	0.915	0.249
Gunnarsdottir et al. (2013)	Pregnant	Highest vs. lowest dairy intake	Iodine status (I/Cr)	0.288	0.161	-0.027	0.604	0.074
Menéndez Torre et al. (2014)	Pregnant	Highest vs. lowest dairy intake	Iodine status (UIC)	0.252	0.166	-0.074	0.578	0.130
Kedir et al. (2014)	Pregnant	Highest vs. lowest milk intake	Iodine status (UIC)	0.217	0.096	0.028	0.405	0.024
Ferreira et al. (2021)	Pregnant	Avg SMD for highest vs. lowest milk, cheese, & yogurt intake	Iodine status (I/Cr)	0.188	0.174	-0.153	0.529	0.281
Johannesen et al. (2024)	Pregnant	Avg SMD for cheese & milk intake vs. no intake	Iodine status (UIC)	0.185	0.088	0.013	0.357	0.036
Perrine et al. (2010)	Pregnant	Dairy intake vs. no intake	Iodine status (UIC)	0.139	0.160	-0.175	0.452	0.386
Ollero et al. (2020)	Pregnant	Highest vs. lowest dairy intake	Iodine status (UIC)	0.131	0.113	-0.090	0.353	0.245
Knight et al. (2017)	Pregnant	Avg SMD for highest cheese, milk & yogurt intake vs. no intake	Iodine status (UIC)	0.113	0.270	-0.416	0.642	0.676
González-Martínez et al. (2021)	Pregnant	Highest vs. lowest dairy intake	Iodine status (UIC)	0.084	0.121	-0.154	0.321	0.489
Opazo et al. (2023)	Pregnant	Highest vs. lowest milk intake	Iodine status (I/Cr)	0.080	0.403	-0.710	0.870	0.843
Blumenthal et al. (2012)	Pregnant	Avg SMD for highest vs. lowest milk & dairy intake	Iodine status (UIC)	-0.084	0.179	-0.435	0.267	0.639
Huang et al. (2020)	Pregnant	Highest vs. lowest dairy intake	Iodine status (UIC)	-0.091	0.167	-0.418	0.237	0.588
Pooled				0.355	0.061	0.236	0.474	0.000

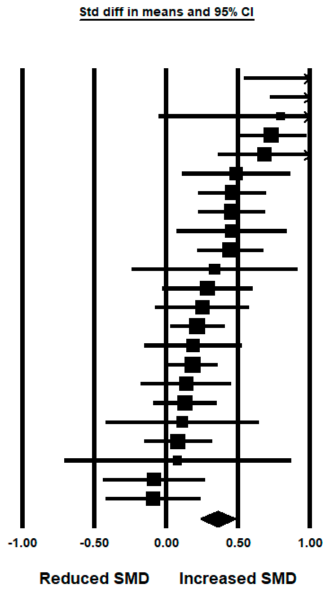

Supplementary Figure S3: Meta-analysis of dairy intake and urinary iodine status using a random-effects model in pregnant women (studies in lactating women not identified) – sensitivity analysis based on method of urinary iodine status assessment (n = 23 publications) [33,34,36–38,41,45,48,50,51,53,55,56,58,59,62,64,65,68–70,78,81]. In this forest plot, each study or stratum is represented by a square indicating the point estimate, with horizontal lines showing the 95% CI. The square size reflects the relative weight of the study or stratum in the analysis. The diamond reflects the pooled estimate. In this meta-analysis, for the five studies in which urinary iodine status was assessed using both UIC and I/Cr, I/Cr data were used instead of UIC as a sensitivity analysis. The association remained statistically significant such that urinary iodine status was significantly greater with higher dairy intake (SMD = 0.355; 95% CI: 0.236, 0.474; $p < 0.001$; $I^2 = 71.38\%$). Avg = average; CI = confidence interval; h = hour; I/Cr = iodine-to-creatinine ratio; SMD = standardized mean difference; Std diff = standardized difference; UIC = urinary iodine concentration; UIE = urinary iodine excretion.
