# Supplementary material for: Dairy Intake and Iodine Status in Pregnant and Lactating Women: A Systematic Review and Meta-Analysis
Source: Nutrients. 2025 Nov 30;17(23):3765. doi: 10.3390/nu17233765 (PMC12693841; doi:10.3390/nu17233765)
Supplement: Supplementary file 1 [file nutrients-17-03765-s001.zip › Fig S4_MA_FishersZ_Dairy & Overall Urine Iodine _SMD_ 25Nov2025.pdf]

Study name	Subgroup	Comparison	Outcome	Statistics for each study				
				Std diff in means	Standard error	Lower limit	Upper limit	p-Value
Johnsen et al. (2025)	Pregnant	Dairy intake (per g/d)	Iodine status (UIC)	0.505	0.182	0.149	0.861	0.005
Perrine et al. (2010)	Pregnant	Dairy intake (per g/d)	Iodine status (UIC)	0.403	0.120	0.167	0.639	0.001
Refaat et al. (2021)	Pregnant	Avg Fisher's Z for cheese, milk & yogurt intakes	Iodine status (24-h UIC)	0.364	0.071	0.225	0.503	0.000
Dineva et al. (2020) (ALSPAC study)	Pregnant	Milk and dairy products intakes (per g/d)	Iodine status (I/Cr)	0.301	0.038	0.226	0.376	0.000
Brantsaeter et al. (2009)	Pregnant	Dairy products intake (per g/d)	Iodine status (24-h UIE)	0.283	0.188	-0.085	0.651	0.132
Stråvik et al. (2021)	Pregnant	Avg Fisher's Z for dairy & cheese intakes	Iodine status (UIC)	0.200	0.060	0.082	0.319	0.001
Dineva et al. (2020) (Generation R study)	Pregnant	Milk and dairy products intakes (per g/d)	Iodine status (I/Cr)	0.170	0.050	0.072	0.269	0.001
Dineva et al. (2020) (INMA study)	Pregnant	Milk and dairy products intakes (per g/d)	Iodine status (I/Cr)	0.160	0.052	0.058	0.262	0.002
Dahl et al. (2018)	Pregnant	Dairy intake (per g/d)	Iodine status (UIC)	0.100	0.120	-0.135	0.336	0.405
Johannesen et al. (2024)	Pregnant	Dairy intake (per g/d)	Iodine status (UIC)	0.026	0.082	-0.135	0.187	0.751
Pooled				0.228	0.037	0.154	0.301	0.000

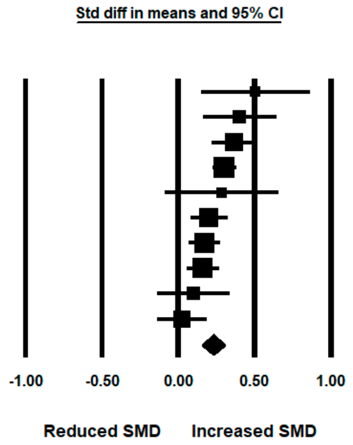

Supplementary Figure S4: Meta-analysis of dairy intake and urinary iodine status in pregnant and lactating women using a random-effects model (Fisher's Z converted from beta and correlation coefficients) (*n* = 8 publications) [39,45,46,55,56,70,73,75]. In this forest plot, each study or stratum is represented by a square indicating the point estimate, with horizontal lines showing the 95% CI. The square size reflects the relative weight of the study or stratum in the analysis. The diamond reflects the pooled estimate. Pooling the results yielded a significantly greater urinary iodine status with higher dairy intake (SMD: 0.228; 95% CI: 0.154, 0.301; $p < 0.001$; $I^2 = 59.495\%$). Avg = average; CI = confidence interval; h = hour; I/Cr = iodine-to-creatinine ratio; SMD = standardized mean difference; Std diff = standardized difference; UIC = urinary iodine concentration; UIE = urinary iodine excretion.
