# Supplementary material for: Dairy Intake and Iodine Status in Pregnant and Lactating Women: A Systematic Review and Meta-Analysis
Source: Nutrients. 2025 Nov 30;17(23):3765. doi: 10.3390/nu17233765 (PMC12693841; doi:10.3390/nu17233765)
Supplement: Supplementary file 1 [file nutrients-17-03765-s001.zip › Fig S5_PB_FishersZ_Dairy & Overall Urine Iodine _SMD_ 25Nov2025.pdf]

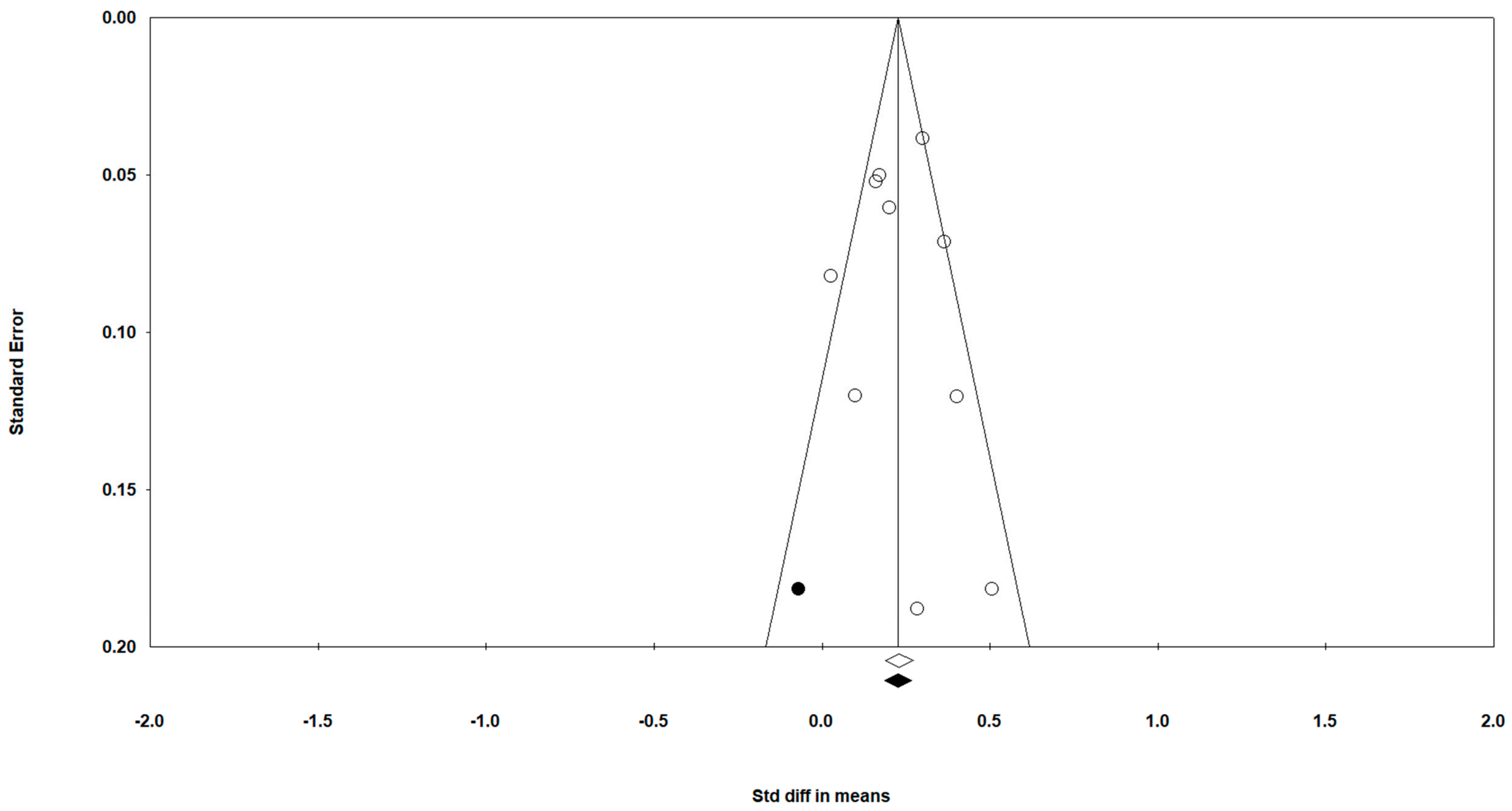

Supplementary Figure S5: Funnel plot of standard error by Std diff in means for dairy intake and urinary iodine status using a random-effects model in pregnant and lactating women (Fisher's Z converted from *beta* and correlation coefficients) (*n* = 8 publications) [39,45,46,55,56,70,73,75]. The trim-and-fill method imputed 1 study missing to the left of the pooled effect; with this study imputed, the SMD remained significant such that urinary iodine status was significantly greater with higher dairy intake (SMD: 0.226; 95% CI: 0.143, 0.292). CI = confidence interval; Std diff = standardized difference; SMD = standardized difference in means.
