# Supplementary material for: Dairy Intake and Iodine Status in Pregnant and Lactating Women: A Systematic Review and Meta-Analysis
Source: Nutrients. 2025 Nov 30;17(23):3765. doi: 10.3390/nu17233765 (PMC12693841; doi:10.3390/nu17233765)
Supplement: Supplementary file 1 [file nutrients-17-03765-s001.zip › Fig S6_sensitivity_FishersZ_Dairy & Overall Urine Iodine _SMD_ 25Nov2025.pdf]

Study name	Subgroup	Comparison	Outcome	Point	Standard error	Lower limit	Upper limit	p-Value
				Statistics with study removed				
Johannesen et al. (2024)	Pregnant	Dairy intake (per g/d)	Iodine status (UIC)	0.248	0.035	0.180	0.316	0.000
Dineva et al. (2020) (INMA study)	Pregnant	Milk and dairy products intakes (per g/d)	Iodine status (I/Cr)	0.240	0.042	0.157	0.322	0.000
Dineva et al. (2020) (Generation R study)	Pregnant	Milk and dairy products intakes (per g/d)	Iodine status (I/Cr)	0.239	0.043	0.154	0.323	0.000
Dahl et al. (2018)	Pregnant	Dairy intake (per g/d)	Iodine status (UIC)	0.237	0.039	0.160	0.314	0.000
Stråvik et al. (2021)	Pregnant	Avg Fisher's Z for dairy & cheese intakes	Iodine status (UIC)	0.233	0.043	0.149	0.318	0.000
Brantsaeter et al. (2009)	Pregnant	Dairy products intake (per g/d)	Iodine status (24-h UIE)	0.226	0.039	0.150	0.303	0.000
Johnsen et al. (2025)	Pregnant	Dairy intake (per g/d)	Iodine status (UIC)	0.218	0.037	0.145	0.291	0.000
Perrine et al. (2010)	Pregnant	Dairy intake (per g/d)	Iodine status (UIC)	0.216	0.038	0.141	0.291	0.000
Dineva et al. (2020) (ALSPAC study)	Pregnant	Milk and dairy products intakes (per g/d)	Iodine status (I/Cr)	0.214	0.041	0.133	0.294	0.000
Refaat et al. (2021)	Pregnant	Avg Fisher's Z for cheese, milk & yogurt intakes	Iodine status (24-h UIC)	0.210	0.038	0.134	0.285	0.000
Pooled				0.228	0.037	0.154	0.301	0.000

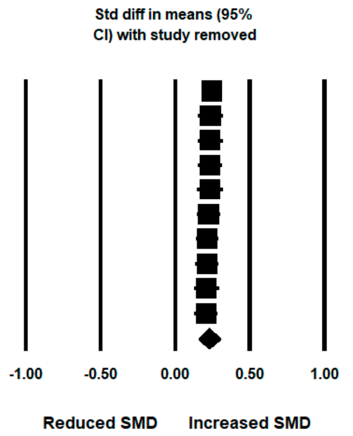

Supplementary Figure S6: Sensitivity (leave-one-out) analysis for dairy intake and urinary iodine status in pregnant and lactating women (Fisher's Z converted from *beta* and correlation coefficients) (n = 8 publications) [39,45,46,55,56,70,73,75]. Each square and horizontal line within each row show the overall pooled estimate and 95% CI, respectively, with the study in the corresponding row left out. Avg = average; CI = confidence interval; h = hour; I/Cr = iodine-to-creatinine ratio; SMD = standardized mean difference; Std diff = standardized difference; UIC = urinary iodine concentration; UIE = urinary iodine excretion.
