# Supplementary material for: Dairy Intake and Iodine Status in Pregnant and Lactating Women: A Systematic Review and Meta-Analysis
Source: Nutrients. 2025 Nov 30;17(23):3765. doi: 10.3390/nu17233765 (PMC12693841; doi:10.3390/nu17233765)
Supplement: Supplementary file 1 [file nutrients-17-03765-s001.zip › Fig S7_PB_Dairy & Iodine deficiency_OR_Adjusted selected data 25Nov2025.pdf]

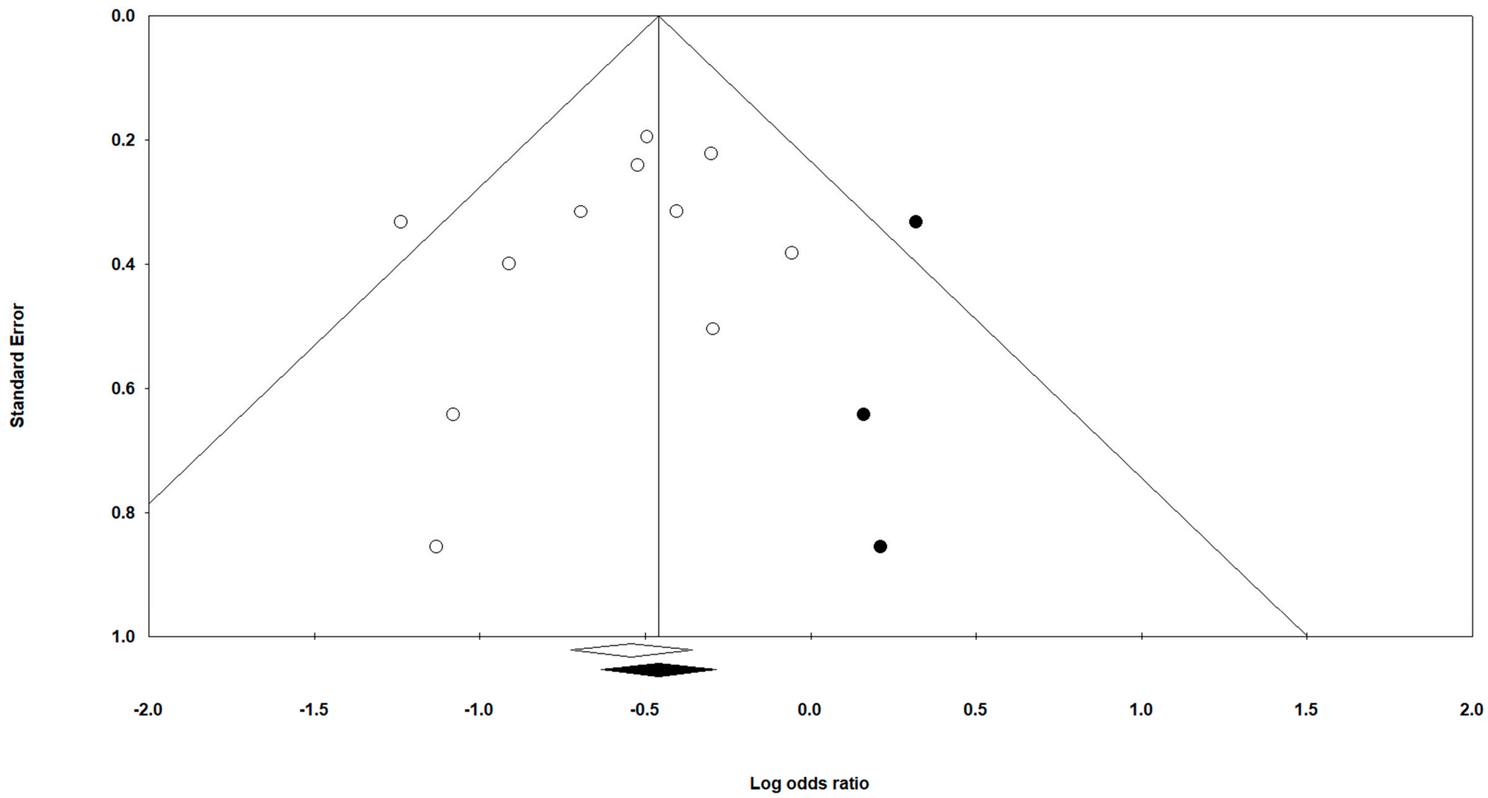

Supplementary Figure S7: Funnel plot of standard error by log OR for dairy intake and odds of iodine deficiency using a random-effects model in pregnant and lactating women (*n* = 12 publications) [32,34,48–50,54,58,65,77,78,80,81]. The trim-and-fill method imputed 3 studies missing to the right of the pooled effect; the bias-adjusted pooled effect remained protective such that there was a significant reduction in the odds of iodine deficiency with higher dairy intake (OR: 0.631; 95% CI: 0.507, 0.784). CI = confidence interval; OR = odds ratio; Std diff = standardized difference.
