# Supplementary material for: Dairy Intake and Iodine Status in Pregnant and Lactating Women: A Systematic Review and Meta-Analysis
Source: Nutrients. 2025 Nov 30;17(23):3765. doi: 10.3390/nu17233765 (PMC12693841; doi:10.3390/nu17233765)
Supplement: Supplementary file 1 [file nutrients-17-03765-s001.zip › Table S1_DMI_Databases 25Nov2025.pdf]

Supplementary Table S1. Electronic databases used to retrieve literature.

Electronic Database	Date Range of Database	
	Original Search	Updated Search
AdisInsight: Trials,	1990 to 12 August 2024	12 August 2024 to 05 March 2025
Allied & Complementary Medicine™	1985 to 12 August 2024	12 August 2024 to 05 March 2025
BIOSIS Previews®	1926 to 12 August 2024	12 August 2024 to 05 March 2025
CAB ABSTRACTS	1910 to 12 August 2024	12 August 2024 to 05 March 2025
Embase®	1947 to 12 August 2024	12 August 2024 to 05 March 2025
Embase Preprints	2013 to 12 August 2024	12 August 2024 to 05 March 2025
Foodline®: SCIENCE	1972 to 2016	N/A
FSTA®	1969 to 12 August 2024	12 August 2024 to 05 March 2025
MEDLINE®	1946 to 12 August 2024	12 August 2024 to 05 March 2025
NTIS: National Technical Information Service	1964 to 12 August 2024	12 August 2024 to 05 March 2025

N/A = not applicable.
