# Supplementary material for: Dairy Intake and Iodine Status in Pregnant and Lactating Women: A Systematic Review and Meta-Analysis
Source: Nutrients. 2025 Nov 30;17(23):3765. doi: 10.3390/nu17233765 (PMC12693841; doi:10.3390/nu17233765)
Supplement: Supplementary file 1 [file nutrients-17-03765-s001.zip › Table S2_DMI_Search strategy 25Nov2025.pdf]

Supplementary Table S2. Keywords used to retrieve literature[a]

Keyword Category	Terms
Keywords used for exposure	ti(iodine* or iodide*)
Keywords used for study population	ti(pregnan* or lactat* or breastfe* or breast-fe* or "breast fe*" or "reproductive age" or "childbearing age" or "child-bearing age" or maternal or mother* or woman or women or female* or nursing)
Keywords used for dairy consumption	ti,ab(*dairy or *milk* or yogurt* or yoghurt* or *cheese* or *cream* or butter* or kefir or skyr or ayran or uunijuusto or viili or ymer or zincica or plant*)
Keywords used to restrict the publication date[b]	PD(>20240811)

[a] For an article to be identified, at least 1 keyword for the exposure (e.g., "iodine") and at least 1 keyword for the study population (e.g., "pregnant") had to appear in the title of the article. Additionally, at least 1 keyword for dairy (e.g., "milk") had to appear either in the title or abstract of the article. For the updated search, a date restriction was imposed such that studies published on or after the date of the original search (12 August 2024) would be identified. No limitations were placed on the language of publication. The asterisk indicates a truncation, so as not to place a limitation on the word ending (e.g., "pregnan*" would result in the identification of "pregnancy" and "pregnant").

[b] The date restriction was imposed only for the updated search.
