# Supplementary material for: Dairy Intake and Iodine Status in Pregnant and Lactating Women: A Systematic Review and Meta-Analysis
Source: Nutrients. 2025 Nov 30;17(23):3765. doi: 10.3390/nu17233765 (PMC12693841; doi:10.3390/nu17233765)
Supplement: Supplementary file 1 [file nutrients-17-03765-s001.zip › Table S3_DMI_KSC_Pregnancy 25Nov2025.pdf]

Supplementary Table S3. Key characteristics of studies conducted in pregnant women (*n* = 42 publications, 42 studies).

Reference<br><br>Quality	Study Design	Country of Conduct	Data Collection		Demographic Data				Dairy Intake Assessment			Outcomes	
			Period	Season	Number of Subjects (n)	Age (y)[a]	GA at Time of AssessmentError! Bookmark not defined.	% With Thyroid Disease	Dietary Intake Tool (Tool)	Assessment Period	Type of Dairy	Outcome Measure	Variables Included in Most Highly Adjusted Model
Aakre et al. [32][b]<br><br>Medium	PC	Norway	September 2011–November 2014	NR	928	30.2 ± 4.8	Range: 9–34 wk[c]	3.2%	SQ FFQ (NR)	Previous 3 mo	All dairy (milk and dairy products)	IS	Maternal age; parity; breastfeeding; time; time*food group; iodine supplements
Adalsteinsdottir et al. [33]<br><br>Medium	CS	Iceland	02 October 2017–28 March 2018	Winter, spring	983	Range: 18–45	Range: 11–14 wk	NR	FFQ (NR)	Previous 3 mo; previous d	All dairy (milk and fermented dairy products)	UIC	Unadjusted
Alvarez-Pedrerol et al. [34]<br><br>Medium	CS	Spain	July 2004–July 2006	Spring, summer, autumn, winter	600	Range: 18–43	Mean (range): 34 (28–40) wk	13%	SQ FFQ (Adapted Willett FFQ)	Previous mo	Milk	UIC	Unadjusted
												IS	GA; maternal weight and age; season and time of extraction; mother's thyroid alterations diagnosed; smoking

Reference<br><br>Quality	Study Design	Country of Conduct	Data Collection		Demographic Data				Dairy Intake Assessment			Outcomes	
			Period	Season	Number of Subjects (n)	Age (y)[a]	GA at Time of AssessmentError! Bookmark not defined.	% With Thyroid Disease	Dietary Intake Tool (Tool)	Assessment Period	Type of Dairy	Outcome Measure	Variables Included in Most Highly Adjusted Model
Bath et al. [37]<br><br>Medium	CS	UK	July–September 2009	Summer	100	32.4 ± 4.7	12 wk	0%	SQ FFQ (NR)	Usual intake	Milk	UIC	Maternal age; supplement use; milk, seafood, and egg intake
											All dairy (cream, yogurt, butter, and cheese)	UIC	Unadjusted
Bath et al. [36]<br><br>High	CS (seasonal results reported prospect-ively)	UK	July 2009–June 2011	Summer, winter	228	30.73 ± 4.14	Recruitment: 12–14 wk<br><br>Follow-up: 20 and 35 wk	0%	FFQ (NR)	Usual intake	Milk; all dairy (type NR)	UIC	Maternal age; age at which education ceased; milk intake; smoking status; consumption of Brazil nuts
												UIC (in seasonal analyses)	BMI at 12 wk; maternal age; season; milk intake; first-order interactions
Blumenthal et al. [38]<br><br>Medium	CS	Australia	November 2007–February 2009	NR	367	32 ± 4.3	Range: 7–11 wk	0%[d]	SQ FFQ (NR)	Usual intake	Milk; dairy products	UIC	Unadjusted

Reference<br><br>Quality	Study Design	Country of Conduct	Data Collection		Demographic Data				Dairy Intake Assessment			Outcomes	
			**Period**	**Season**	**Number of Subjects (n)**	**Age (y)[a]**	**GA at Time of AssessmentError! Bookmark not defined.**	**% With Thyroid Disease**	**Dietary Intake Tool (Tool)**	**Assessment Period**	**Type of Dairy**	**Outcome Measure**	**Variables Included in Most Highly Adjusted Model**
Brantsaeter et al. [39][e]<br><br>Medium	CS	Norway	15 January 2003–01 February 2004	Spring, summer, autumn, winter	119	Mean (range): 31 (23–44)	Range: 17–18 wk	NR	SQ FFQ (MoBa FFQ); food diary	First 4–5 mo of pregnancy; previous 4 d	All dairy (milk beverages, cheese, yogurt, milk in foods, butter); milk; cheese; yogurt; butter	UIC	Total energy intake; time between assessment methods
												% contr. to TDII	N/A
Brantsaeter et al. [40]<br><br>Medium	CS	Norway	2002–2008	NR	61,904	NR	Range: 17–22 wk	NR	SQ FFQ (MoBa FFQ)	First 4–5 mo of pregnancy	Milk and yogurt	IS	Maternal age; parity; education; marital status; smoking; BMI; household income; total energy intake
												TDII	Unadjusted
												% contr. to TDII	N/A

Reference<br><br>Quality	Study Design	Country of Conduct	Data Collection		Demographic Data				Dairy Intake Assessment			Outcomes	
			Period	Season	Number of Subjects (n)	Age (y)[a]	GA at Time of AssessmentError! Bookmark not defined.	% With Thyroid Disease	Dietary Intake Tool (Tool)	Assessment Period	Type of Dairy	Outcome Measure	Variables Included in Most Highly Adjusted Model
Cannas et al. [41]<br><br>Medium	CS	Cyprus	May–June 2019	Summer	128	Range: 18–44	Range: 10–13 wk	0%	FFQ (NR)	Usual intake	All dairy (cheese, cream, yogurt, butter, and dairy desserts); milk	UIC	BMI; kelp and/or iodine-containing supplements; dairy products; eggs; seafood
Castilla et al. [42][f]<br><br>High	PC	Spain	November 2003–February 2008	NR	291	32 ± 4.1	Median (range): 12.6 (8.9–16.6) wk	NR	SQ FFQ (NR)	First half of pregnancy	All dairy (type NR)	% contr. to TDII	N/A
Charlton et al. [43]<br><br>Low	CS	Australia	2011; 2012	NR	Total: 255 (2011: 146; 2012: 109)	2011: 28 ± 5.0<br><br>2012: 29.6 ± 5.8	Any GA	0%	FFQ (NR)	Previous mo	Milk and dairy	% contr. to TDII	N/A
Condo et al. [44]<br><br>Low	PC	Australia	August 2011–December 2012	NR	783	30.5 ± 5	Recruitment: 16.3 ± 2 wk<br><br>Follow-up: 28 wk	0%	FFQ (Iodine FFQ)	Usual intake	All dairy (type NR); milk	% contr. to TDII	N/A

Reference<br><br>Quality	Study Design	Country of Conduct	Data Collection		Demographic Data				Dairy Intake Assessment			Outcomes	
			**Period**	**Season**	**Number of Subjects (n)**	**Age (y)[a]**	**GA at Time of AssessmentError! Bookmark not defined.**	**% With Thyroid Disease**	**Dietary Intake Tool (Tool)**	**Assessment Period**	**Type of Dairy**	**Outcome Measure**	**Variables Included in Most Highly Adjusted Model**
Dahl et al. [45]<br><br>Medium	CS	Norway	September 2011–October 2012	Spring, summer, autumn, winter	954	Median (range): 30 (17–43)	Median (range): 25 (9–34) wk	NR	SQ FFQ (NR)	Previous 3 mo	All dairy (type NR)	UIC	Supplement use; maternal residence
Dineva et al. [46]; ALSPAC<br><br>Medium	CS	UK	01 April 1991–31 December 1992	Summer, winter	2852	28.7 ± 4.5	≤18 wk	0%	FFQ (NR)	Usual intake	All dairy (milk and cheese)	UIC	Energy; GA; age; pre-pregnancy BMI; ethnicity; parity; smoking status; alcohol consumption; education; home ownership; crowding index; family adversity index; life event score; marital status; child’s sex

Reference<br><br>Quality	Study Design	Country of Conduct	Data Collection		Demographic Data				Dairy Intake Assessment			Outcomes	
			Period	Season	Number of Subjects (n)	Age (y)[a]	GA at Time of AssessmentError! Bookmark not defined.	% With Thyroid Disease	Dietary Intake Tool (Tool)	Assessment Period	Type of Dairy	Outcome Measure	Variables Included in Most Highly Adjusted Model
Dineva et al. [46]; Generation R<br><br>Medium	CS	Netherlands	April 2002–January 2006	NR	2254	29.9 ± 5.0	≤18 wk	0%	SQ FFQ (NR)	Previous 3 mo	All dairy (milk, yogurt, cheese, cream, and dairy desserts)	UIC	Energy; GA; age; pre-pregnancy BMI; ethnicity; parity; smoking status; alcohol consumption; education; net household income; marital status; child's sex
Dineva et al. [46]; INMA[f]<br><br>Medium	CS	Spain	November 2003–January 2008	NR	1460	31.4 ± 4.1	≤18 wk	0%	SQ FFQ (NR)	Usual intake	All dairy (milk, yogurt, cheese, cream, and dairy desserts)	UIC	Energy; GA; age; pre-pregnancy BMI; ethnicity; parity; smoking status; alcohol consumption; education; living with a partner; child's sex
Farha et al. [47]<br><br>Medium	CS	India	January–December 2019	Winter	110	23.87 ± 3.91	First trimester	0%	FFQ (NR)	Usual intake	Milk	IS	Unadjusted
	CS	Portugal		NR	468	32 ± 5		0%	FFQ	Usual intake		UIC	Unadjusted

Reference<br><br>Quality	Study Design	Country of Conduct	Data Collection		Demographic Data				Dairy Intake Assessment			Outcomes	
			Period	Season	Number of Subjects (n)	Age (y)[a]	GA at Time of AssessmentError! Bookmark not defined.	% With Thyroid Disease	Dietary Intake Tool (Tool)	Assessment Period	Type of Dairy	Outcome Measure	Variables Included in Most Highly Adjusted Model
Ferreira et al. [48]<br><br>Medium			April 2018–April 2019				Median (range): 12 (10–13) wk		(NR)		Milk; yogurt; cheese	IS	Age; BMI; education level; smoking habits
González-Martínez et al. [50][g]<br><br>Medium	PC	Spain	May–June 2017	Spring, summer	318	34.1 ± 5.45	Recruitment: 7.19 ± 2 wk<br><br>Follow-up: 25.5 ± 2.6 wk	0%[h]	Question-naire (NR)[i]	Usual intake	All dairy (milk, yogurt, and cheese); milk; yogurt; cheese	UIC	Unadjusted
												IS	Iodized salt; iodine supplement
González-Martínez et al. [49]<br><br>Medium	PC	Spain	May–June 2017	Spring, summer	241	34.1 ± 5.1	Recruitment: 7.19 ± 2 wk<br><br>Follow-up: wk 14 to second trimester	0%[h]	Question-naire (NR)[j]	Usual intake	All dairy (milk, yogurt, and cheese); milk; yogurt; cheese	IS	Iodized salt; dairy products (daily servings of milk, yogurt, and cheese); iodine supplements
Gunnarsdottir et al. [51]<br><br>Medium	CS	Iceland	November 2007–February 2009	Winter	162	30.5 ± 5.5	Second to third trimester	NR	SQ FFQ (NR)	Previous 3 mo	All dairy (milk and cheese)	UIC	Unadjusted

Reference<br><br>Quality	Study Design	Country of Conduct	Data Collection		Demographic Data				Dairy Intake Assessment			Outcomes	
			**Period**	**Season**	**Number of Subjects (n)**	**Age (y)[a]**	**GA at Time of AssessmentError! Bookmark not defined.**	**% With Thyroid Disease**	**Dietary Intake Tool (Tool)**	**Assessment Period**	**Type of Dairy**	**Outcome Measure**	**Variables Included in Most Highly Adjusted Model**
Huang et al. [53]<br><br>Medium	CS	Taiwan	January–October 2018	Winter	257	33.9 ± 4.3	Any GA	NR	FFQ (Iodine Nutrition Question-naire)	Usual intake during gestation	All dairy (milk, cheese, yogurt, and cream)	UIC	Unadjusted
Johannesen et al. [55]<br><br>Medium	CS	Denmark	June 2020–April 2022	Spring, summer, autumn, winter	647	30.4 ± 5	21.4 ± 3.5 wk	3.4%	FFQ (NR)	Previous 7 d; yesterday/ today	Milk; cheese; all dairy (milk and cheese)	UIC	Unadjusted
Johnsen et al. [56]<br><br>Low	CS	Norway	June 2017; June 2021; March 2021–2022	NR	131	28 ± 2.7	15 ± 7.3 wk	0.8%	FFQ (NR)	Previous wk	All dairy (milk, yogurt, sour cream, and cheese)	UIC	Iodine supplementation; lean fish consumption; oily fish consumption
												% contr. to TDII	NA
Kedir et al. [58]	CS	Ethiopia		Summer	435	27 ± 5.9	25.8 ± 5.8 wk	NR		Usual intake	Milk	UIC	Unadjusted

Reference<br><br>Quality	Study Design	Country of Conduct	Data Collection		Demographic Data				Dairy Intake Assessment			Outcomes	
			Period	Season	Number of Subjects (n)	Age (y)[a]	GA at Time of AssessmentError! Bookmark not defined.	% With Thyroid Disease	Dietary Intake Tool (Tool)	Assessment Period	Type of Dairy	Outcome Measure	Variables Included in Most Highly Adjusted Model
Medium			16–29 March 2012						FFQ (Ethiopian Health and Demo-graphic Survey)			IS	Age; education; family size; previous pregnancies; trimester of pregnancy; possessed milk cows; prenatal visit; cabbage consumption; household salt
Knight et al. [59]<br><br>Medium	CS	UK	NR	Summer, winter	308	31 ± 5	35 ± 8.8 wk	0%	FFQ (NR)	Usual intake	*Milk; yogurt; cheese*	UIC	Unadjusted
Lean et al. [60]<br><br>Medium	CS	India	May 2004–July 2006	NR	132 (wk 17); 151 (wk 34)	22.6 ± 3.7	17 and 34 wk	0%	FFQ (NR)	Usual intake	Milk; all dairy (ghee, curd, butter, and cheese)	UIC	SLI score; maternal education
												IS	NR

Reference<br><br>Quality	Study Design	Country of Conduct	Data Collection		Demographic Data				Dairy Intake Assessment			Outcomes	
			Period	Season	Number of Subjects (n)	Age (y)[a]	GA at Time of AssessmentError! Bookmark not defined.	% With Thyroid Disease	Dietary Intake Tool (Tool)	Assessment Period	Type of Dairy	Outcome Measure	Variables Included in Most Highly Adjusted Model
Liu et al. [61]<br><br>Medium	CS	China	January–June 2014	Summer, winter	142	*27.05* (SD NR)	*22.4* wk	0%	NR (NR)	NR	Milk	IS	Water iodine area; per capita annual household income; dietary habits; habit of using cooking salt; frequency of eating cruciferous foods; eating marinated foods; eating meat; TSH concentration; age
McMullan et al. [62]<br><br>Low	PC	UK	July 2014–March 2015	Summer, winter	241	30.3 ± 5.4	Mean (range): 11 (9–17) wk	0%	SQ FFQ (NR)	Usual intake	Milk; yogurt	UIC	Unadjusted
Melero et al. [63]<br><br>Medium	CS	Spain	January 2015–December 2017	NR	2523	32.64 ± 5.19	Range: 9–12 wk	0%[k]	SQ FFQ (Adapted DNCT)	Usual intake	All dairy (type NR)[l]	IS	Age; parity; smoking
Menéndez Torre et al. [64]<br><br>Medium	CS	Spain	May–June 2013	Summer	173	32.8 ± 6.9	7 ± 2 wk	NR	Structured recall (NR)	Usual intake	All dairy (milk, cheese, and yogurt)	UIC	Unadjusted

Reference<br><br>Quality	Study Design	Country of Conduct	Data Collection		Demographic Data				Dairy Intake Assessment			Outcomes	
			**Period**	**Season**	**Number of Subjects (n)**	**Age (y)[a]**	**GA at Time of AssessmentError! Bookmark not defined.**	**% With Thyroid Disease**	**Dietary Intake Tool (Tool)**	**Assessment Period**	**Type of Dairy**	**Outcome Measure**	**Variables Included in Most Highly Adjusted Model**
Mian et al. [65]<br><br>Low	CS	Italy	April 2006–July 2007	NR	322	Range: 15–46	35 ± 8.8 wk	0%	FFQ (NR)	Usual intake	Milk; cheese; yogurt	UIC	Unadjusted
											Milk	IS	Unadjusted
Ollero et al. [68]<br><br>Medium	CS	Spain	2014–2016	NR	400	33.4 ± 4.1	Mean (range): 10 (9–12) wk	0%	Iodine consump-tion question-naire (NR)	Usual intake	All dairy (milk, cheese, and yogurt)	UIC	Unadjusted
Opazo et al. [69][q]<br><br>High	CS	Chile	NR	NR	25	30.4 ± 1.1	Mean ± SEM: 22.8 ± 1.2 wk	0%	24-h recall (NR)	Previous d	Milk	UIC	Unadjusted
												TDII	Unadjusted
												% contr. to TDII	NA
Perrine et al. [70]<br><br>Medium	CS	US	2001–2006	NR	326	Mean (range): 27 (15–39)	Any GA	0%	24-h recall (NR)	Previous d	All dairy (type NR)	UIC	Age; ethnicity; education; grain intake; salt and supplement use; trimester
Refaat et al. [73]<br><br>Medium	CS	Saudi Arabia	February 2018–September 2019	NR	810	28.2 ± 5	Any GA (n=270 per trimester)	32.8%[m]	SQ FFQ (Glasgow Iodine Food Frequency Question-naire)	Usual intake	Yogurt; milk; cheese	UIC	Unadjusted

Reference<br><br>Quality	Study Design	Country of Conduct	Data Collection		Demographic Data				Dairy Intake Assessment			Outcomes	
			Period	Season	Number of Subjects (n)	Age (y)[a]	GA at Time of AssessmentError! Bookmark not defined.	% With Thyroid Disease	Dietary Intake Tool (Tool)	Assessment Period	Type of Dairy	Outcome Measure	Variables Included in Most Highly Adjusted Model
Silva et al. [74]<br><br>Low	CS	Brazil	September 2018–April 2021	Spring, summer, autumn, winter	2247	27 ± 6	Any GA	0%	24-h recall (NR)	Previous d	All dairy (milk, yogurt, and cream, cheese); milk; yogurt	% contr. to TDII	NA
Stråvik et al. [75][b]<br><br>Low	CS	Sweden	February 2015–March 2018	NR	604	Median (90% CI): 30 (23, 39)	FFQ (Mean [range]): 34 (32–40) wk<br><br>UIC (Mean [90%CI]): 29 (27–32) wk	0%	FFQ (Meal-Q)	30–34 wk GA	All dairy (milk, yogurt, sour milk, and cream); milk; cheese; yogurt	UIC	Unadjusted
Threapleton et al. [76][b]<br><br>High	PC	UK	August 2016–October 2017	Summer, winter[n]	246	Median (IQR): 31 (27–34)	Recruitment: 13.9 ± 1.1 wk<br><br>Follow-up visits: 26 and 36 wk	NR[o]	24-h recall (myfood24)[p]	Previous d	All dairy (milk, cream, butter, cheese, and yogurt)	% contr. to TDII	NA
	CS	Spain	2008–2009	NR	970	30.6 ± 4.6	<13 wk	0%	FFQ	Usual intake	Milk	UIC	Unadjusted

Reference<br><br>Quality	Study Design	Country of Conduct	Data Collection		Demographic Data				Dairy Intake Assessment			Outcomes	
			Period	Season	Number of Subjects (n)	Age (y)[a]	GA at Time of AssessmentError! Bookmark not defined.	% With Thyroid Disease	Dietary Intake Tool (Tool)	Assessment Period	Type of Dairy	Outcome Measure	Variables Included in Most Highly Adjusted Model
Torres et al. [78]<br><br>High									(NR)			IS	Daily milk; iodized salt
Vandevijvere et al. [80]<br><br>Medium	CS	Belgium	September 2010–June 2011	Spring, summer, autumn, winter	1311	28.5 ± 5.1	22.2 ± 12.5 wk	2.7%	Question-naire (NR)	Usual intake	All dairy (milk and dairy drinks)	UIC	Age; trimester; region; BMI; smoking; alcohol consumption; iodine supplement use; iodized household salt use; bread consumption; fish consumption; education level; ethnicity; parity
												IS	Age; trimester; region; BMI; smoking; alcohol consumption; iodine supplement use; iodized household salt use; bread consumption; Fish consumption; education level; ethnicity; parity

Reference<br><br>Quality	Study Design	Country of Conduct	Data Collection		Demographic Data				Dairy Intake Assessment			Outcomes	
			Period	Season	Number of Subjects (n)	Age (y)[a]	GA at Time of AssessmentError! Bookmark not defined.	% With Thyroid Disease	Dietary Intake Tool (Tool)	Assessment Period	Type of Dairy	Outcome Measure	Variables Included in Most Highly Adjusted Model
Veisa et al. [81]<br><br>Medium	CS	Latvia	2014	NR	129	Range: 17–40	Range: 6–8 wk	0%	Question-naire (NR)	Usual intake	All dairy (milk and dairy products)	IS	Unadjusted
												UIC	NA
Wu et al. [82]<br><br>Low	CS	China	May–September 2020	Summer	886	30 ± 4	Any GA	0%	FFQ (NR)	Usual intake	All dairy (milk and dairy products)	% contr. to TDII	NA
Zhao et al. [77]<br><br>Low	CS	China	March–October 2018	Summer, winter	1400	29.08 ± 4.55	Any GA	5.5%	Question-naire (NR)	Previous 7 d	Milk; yogurt	UIC	Unadjusted
												IS	Unadjusted

ALSPAC = Avon Longitudinal Study of Parents and Children cohort; BMI = body mass index; CI = confidence interval; contr. = contribution; CS = cross-sectional; d = day(s); DNCT = Diabetes Nutrition and Complication Trial Questionnaire; FFQ = food frequency questionnaire; GA = gestational age; h = hour(s); I/Cr = urinary iodine-to-creatinine ratio; INMA = INfancia y Medio Ambiente cohort; IQR = interquartile range; IS = iodine sufficiency; mo = month(s); MoBA = Norwegian Mother and Child Cohort Study; N/A = not available; NR = not reported; PC = prospective cohort; SD = standard deviation; SLI = Standard of Living Index; SQ = semi quantitative; TDII = total dietary iodine intake; TSH = thyroid-stimulating hormone; UIC = urinary iodine concentration; UK = United Kingdom; wk = week(s); y = year(s).

[a] The values presented are mean ± SD, unless otherwise reported. Italicized numbers were calculated by the review authors.
[b] These studies were prospective in design in which women were recruited during pregnancy and analyzed multiple times during pregnancy and during the postpartum period.
[c] UIC was calculated using generalized estimating equations across all timepoints to 18 mo postpartum.
[d] A family history of thyroid disease (familial or self-reported) was present in 20% of participants.
[e] The publication by Brantsaeter et al. [38] was a validation study for the FFQ using a subgroup of the cohort that was reported in the publication by Brantsaeter et al. [39].
[f] The publication by Castilla et al. [41] is a subgroup analysis of the INMA study in which a subset of participants completed 2 dietary assessments before and after pregnancy. The data from Castilla et al. [41] was not pooled in the meta-analysis due to overlap in data from the publication by Dineva et al. [45] which includes data from the whole cohort.

[g] As mentioned, Gonzalez-Martinez et al. [49] and Gonzalez-Martinez et al. [48] were conducted on the same population, with the 2021 report [49] providing first trimester data and the 2023 report [48] providing second trimester data.

[h] At recruitment, women had no symptoms/signs or history of thyroid disorders or iodine treatment within the last 3 mo. During the study, a subset of the cohort was assessed for thyroid autoimmunity (n=152) with 39 subjects presenting with positive thyroid autoimmunity.

[i] Administered only at first visit (i.e., first trimester).

[j] Administered only at first visit (i.e., first trimester).

[k] At recruitment, women had no symptoms/signs or history of thyroid disorders or iodine treatment within the last 3 mo. During the study, 692 subjects had gestational subclinical hypothyroidism and 488 had hypothyroxinemia.

[l] Dairy consumption was further stratified and analyzed according to fat content (fat-free vs. low-fat vs. full-fat) and according to fortification.

[m] At recruitment, women had no symptoms/signs or history of thyroid disorders or chronic and autoimmune diseases. They were diagnosed with thyroid disorders during the study.

[n] The authors accounted for seasonal variation in their analyses but did not provide detailed data or breakdowns by season.

[o] Women were excluded if they had previous diagnosis or a first-degree relative with history of a thyroid condition. 36% of the women had a palpable goiter during the study.

[p] Administered at each study visit.

[q] This study provided raw data for 26 participants. As one participant was diagnosed with a thyroid disorder during the study, we first excluded this participant and then ran the initial analyses to obtain relative results for UIC, I/Cr, dietary iodine intake, overall iodine status, and the contribution of dairy to iodine status. In this study, dairy was reported as liquid milk intake and powdered milk intake; to calculate the overall milk (as dairy) intake, we converted powdered milk to liquid milk equivalents using international conversion estimates (available at: https://fdc.nal.usda.gov/food-details/170876/measures).
