# Supplementary material for: Dairy Intake and Iodine Status in Pregnant and Lactating Women: A Systematic Review and Meta-Analysis
Source: Nutrients. 2025 Nov 30;17(23):3765. doi: 10.3390/nu17233765 (PMC12693841; doi:10.3390/nu17233765)
Supplement: Supplementary file 1 [file nutrients-17-03765-s001.zip › Table S4_DMI_KSC_Lactating 25Nov2025.pdf]

Supplementary Table S4. Key characteristics of studies conducted in lactating women (n = 9 publications, 9 studies).[a]

Reference	Quality	Country of Conduct	Data Collection		Demographic Data				Dairy Intake Assessment			Outcomes	
			Period	Season	Number of Subjects (n)	Age (y)[b]	PP Assessment Period(s)[b]	% With Thyroid Disease	Dietary Intake Tool (Tool)	Assessment Period	Type of Dairy[c]	Outcome Measure	Variables Included in Most Highly Adjusted Model
Axford et al. [35]	Medium	Australia	NR	NR	60	32 ± 3.9	2.6 ± 1.0 mo	NR	FFQ (NR)	Usual intake	All dairy (milk, cheese, yogurt, ice cream, and custard)	% contr. to TDII	N/A
Henjum et al. [52]	Low	Norway	October 2016–December 2016	Winter	175	32 ± 4.2	Up to 6 mo	6.3%	FFQ (NR)	Usual intake	All dairy (milk and yogurt)	% contr. to TDII	N/A
									24-h dietary recall	Previous 24 h	All dairy (milk and yogurt)	% contr. to TDII	N/A
Huang et al. [54]	Medium	Taiwan	August 2021–February 2023	NR	71	34 ± 4.1	44.8 ± 9.4 d	0%	FFQ (Iodine Nutrition Questionnaire)	Usual intake	All dairy (milk, cheese, yogurt, butter, and ice cream)	BMIC	Variables NR
												IS	Unadjusted
Jorgensen et al. [57]	Medium	Australia	February 2014–August 2015	NR	55	31.4 ± 4.7	38.5 ± 5.5 d	0%	Structured food checklist (NR)	Usual intake	Milk	BMIC	Other food groups; iodine supplements; iodized salt; ethnicity; education

Reference	Quality	Country of Conduct	Data Collection		Demographic Data				Dairy Intake Assessment			Outcomes	
			Period	Season	Number of Subjects (n)	Age (y)[b]	PP Assessment Period(s)[b]	% With Thyroid Disease	Dietary Intake Tool (Tool)	Assessment Period	Type of Dairy[c]	Outcome Measure	Variables Included in Most Highly Adjusted Model
Moon et al. [66]	Medium	South Korea	NR	NR	50	28 (SD NR)	2–5 d; 4 wk	NR	24-h dietary recall (NR)	Previous 24 h	Milk	BMIC	Unadjusted
												TDII	Unadjusted
Nazeri et al. [67]	Low[d]	Iran	NR	NR	84	28.2 (4.5)	3–5 (baseline), 7, 10, and 14 d, and 1 mo	0%	NR	NR	Milk (fortified with 150 μg/day iodine)	UIC	Mothers' occupation; gravidity; iodine supplements; iodized salt; baseline UIC
												BMIC	Mothers' occupation; gravidity; iodine supplements; iodized salt; baseline BMIC
Petersen et al. [71]	Low	Iceland	December 2014–June 2017	NR	60	EBF: 30.2 ± 4.4<br>PBF: 30.6 ± 5.3	Up to 5 mo	NR	FFQ (NR)	Usual intake	All dairy (NR)	BMIC	Unadjusted
												UIC	Unadjusted
Prpić et al. [72]	Low	Croatia	2014–2016	NR	133	31.5 ± 4.7	2–24 wk	0%	Questionnaire (NR)	Previous month	Milk and yogurt; cheese	BMIC	Unadjusted
												UIC	Unadjusted

Reference	Quality	Country of Conduct	Data Collection		Demographic Data				Dairy Intake Assessment			Outcomes	
			Period	Season	Number of Subjects (n)	Age (y)[b]	PP Assessment Period(s)[b]	% With Thyroid Disease	Dietary Intake Tool (Tool)	Assessment Period	Type of Dairy[c]	Outcome Measure	Variables Included in Most Highly Adjusted Model
Trabzuni et al. [79]	Low	Saudi Arabia	December 1995–February 1996	NR	104	Range: 17–42	6 to 12 wk	NR	FFQ (NR)	Usual intake	All dairy (milk, cheese, and yogurt)	BMIC	Unadjusted
												% contr. to TDII	N/A
												UIC	Unadjusted

BF = breastfeeding; BM = breast milk; BMIC = breast milk iodine concentration; contr. = contribution; EBF = exclusively breastfeeding; FFQ = food frequency questionnaire; h = hour(s); IS = iodine sufficiency; mo = month(s); N/A = not applicable; NR = not reported; PBF = partially breastfeeding; PP = postpartum; SD = standard deviation; TDII = total dietary iodine intake; UIC = urinary iodine concentration; wk = week(s); y = year(s).

[a] All studies were cross-sectional observational in design except for the study by Nazeri et al. [66] which was a randomized controlled trial.
[b] The values presented are mean ± SD or mean (range), unless otherwise reported.
[c] The type of dairy included in the analysis of iodine levels in urine or BM varied across the studies. Semi-colons in this column represent the dairy products for which separate analyses were presented.
[d] According to the Risk of Bias 2 tool for randomized controlled trials, this study was rated as having a high risk of bias.
