# Supplementary material for: Dairy Intake and Iodine Status in Pregnant and Lactating Women: A Systematic Review and Meta-Analysis
Source: Nutrients. 2025 Nov 30;17(23):3765. doi: 10.3390/nu17233765 (PMC12693841; doi:10.3390/nu17233765)
Supplement: Supplementary file 1 [file nutrients-17-03765-s001.zip › Table S5_DMI_QA Summary 25Nov2025.pdf]

Supplementary Table S5. Summary of study quality appraisals (n = 51).

Reference<br>Study Population	Quality Score	Quality Rating[a]	Key Limitations[b]
**Assessment of prospective cohort studies using the OSQE tool[c]**			
Aakre et al. [31]<br><br>Pregnant/lactating	8/15	Medium	• Sample was not optimal for both internal validity and representativeness<br>• Subjects with thyroid disease were not explicitly excluded[1]<br>• Assessment of the exposure was not valid for the study population<br>• Characterization of exposure was not optimal<br>• Assessor was not blinded to the exposure<br>• Methodology for handling missing data not reported<br>• Effect modification analysis not conducted
Castilla et al. [41]<br><br>Pregnant	9/15	High	• Sample was not optimal for both internal validity and representativeness<br>• Subjects with thyroid disease were not explicitly excluded[1]<br>• Exposure was not characterized<br>• Methodology for handling missing data not reported<br>• Relevant confounders not controlled for in statistical analysis<br>• Effect modification analysis not conducted
Condo et al. [43]<br><br>Pregnant	7/15	Low	• Sample was not optimal for both internal validity and representativeness<br>• Subjects with thyroid disease were not explicitly excluded[2]<br>• Assessment of the exposure was not valid for the study population<br>• Exposure was not characterized<br>• Blinding of the assessor to the exposure not reported<br>• Methodology for handling missing data not reported<br>• Relevant confounders not controlled for in statistical analysis<br>• Effect modification analysis not conducted

[1] Thyroid disease was not an inclusion criterion.

[2] Subjects with thyroid disease at baseline were excluded; however, the development of thyroid disease during the study was not assessed.

Reference<br>Study Population	Quality Score	Quality Rating[a]	Key Limitations[b]
González-Martínez et al. [48]<br><br>Pregnant	8/15	Medium	• Sample was not optimal for both internal validity and representativeness<br>• Subjects with thyroid disease were not explicitly excluded[1]<br>• Assessment of the exposure was not valid for the study population<br>• Exposure was not characterized<br>• Assessor was not blinded to the exposure<br>• Methodology for handling missing data not reported<br>• Effect modification analysis not conducted
McMullan et al. [61]<br><br>Pregnant	7/15	Low	• Sample was not optimal for both internal validity and representativeness<br>• Subjects with thyroid disease were not explicitly excluded[2]<br>• Assessment of the exposure was not valid for the study population<br>• Blinding of the assessor to the exposure not reported<br>• High loss-to-follow up for dairy exposure assessment<br>• Methodology for handling missing data not reported<br>• Relevant confounders not controlled for in statistical analysis<br>• Effect modification analysis not conducted
Threapleton et al. [74]<br><br>Pregnant/lactating	10/15	High	• Sample was not optimal for both internal validity and representativeness<br>• Subjects with thyroid disease were not explicitly excluded[1]<br>• Assessment of the exposure was not valid for the study population<br>• Characterization of exposure was not optimal<br>• Relevant confounders not controlled for in statistical analysis
**Assessment of cross-sectional studies using the OSQE tool**			
Adalsteinsdottir et al. [32]<br><br>Pregnant	5/9	Medium	• Sample was not optimal for both internal validity and representativeness<br>• Subjects with thyroid disease were not explicitly excluded[2]<br>• Relevant confounders not controlled for in statistical analysis<br>• Methodology for handling missing data not reported<br>• Effect modification analysis not conducted

[1] Thyroid disease was assessed during the study and all subjects were included, irrespective of thyroid disease status
[2] Thyroid disease was not an exclusion criterion

**Reference**<br>**Study Population**	**Quality Score**	**Quality Rating[a]**	**Key Limitations[b]**
Alvarez-Pedrerol et al. [33]<br><br>Pregnant	5/9	Medium	• Sample was not optimal for both internal validity and representativeness<br>• Subjects with thyroid disease were not explicitly excluded[1]<br>• Assessment of the exposure was not valid for the study population<br>• Conflict of interest statement not provided<br>• Relevant confounders not controlled for in statistical analysis
Axford et al. [34]<br><br>Lactating	4/9	Medium	• Sample was not optimal for both internal validity and representativeness<br>• Subjects with thyroid disease were not explicitly excluded[1]<br>• Assessment of the exposure was not valid for the study population<br>• Conflict of interest statement not provided<br>• Relevant confounders not controlled for in statistical analysis<br>• Effect modification analysis not conducted
Bath et al. [36]<br><br>Pregnant	4/9	Medium	• Sample was not optimal for both internal validity and representativeness<br>• Subjects with thyroid disease were not explicitly excluded[2]<br>• Assessment of the exposure was not valid for the study population<br>• **Characterization of exposure was not optimal[d]**<br>• Relevant confounders not controlled for in statistical analysis<br>• Effect modification analysis not conducted
Blumenthal et al. [37]<br><br>Pregnant	4/9	Medium	• Sample was not optimal for both internal validity and representativeness<br>• Subjects with thyroid disease were not explicitly excluded[2]<br>• Validity of the exposure assessment cannot be determined<br>• Conflict of interest statement not provided<br>• Relevant confounders not controlled for in statistical analysis<br>• Effect modification analysis not conducted

[1] Thyroid disease was not an exclusion criterion

[2] Subjects with thyroid disease at baseline were excluded; however, the development of thyroid disease during the study was not assessed.

Reference<br>Study Population	Quality Score	Quality Rating[a]	Key Limitations[b]
Brantsaeter et al. [38]<br><br>Pregnant	5/9	Medium	• Sample was not optimal for both internal validity and representativeness<br>• Subjects with thyroid disease were not explicitly excluded[1]<br>• Exposure was not characterized<br>• Conflict of interest statement not provided<br>• Relevant confounders not controlled for in statistical analysis
Brantsaeter et al. [39]<br><br>Pregnant	5/9	Medium	• Sample was not optimal for both internal validity and representativeness<br>• Subjects with thyroid disease were not explicitly excluded[1]<br>• Methodology for handling missing data not reported<br>• Potential conflict of interest based on author affiliations<br>• Relevant confounders not controlled for in statistical analysis
Cannas et al. [40]<br><br>Pregnant	4/9	Medium	• Sample was not optimal for both internal validity and representativeness<br>• Subjects with thyroid disease were not explicitly excluded[1]<br>• Assessment of the exposure was not valid for the study population<br>• Relevant confounders not controlled for in statistical analysis<br>• Methodology for handling missing data not reported<br>• Effect modification analysis not conducted
Charlton et al. [42]<br><br>Pregnant	3/9	Low	• Sample was not optimal for both internal validity and representativeness<br>• Subjects with thyroid disease were not explicitly excluded[1]<br>• Assessment of the exposure was not valid for the study population<br>• Exposure was not characterized<br>• Relevant confounders not controlled for in statistical analysis<br>• Methodology for handling missing data not reported<br>• Effect modification analysis not conducted

[1] Subjects with thyroid disease at baseline were excluded; however, the development of thyroid disease during the study was not assessed

**Reference**<br>**Study Population**	**Quality Score**	**Quality Rating[a]**	**Key Limitations[b]**
Dahl et al. [44]<br><br>Pregnant	5/9	Medium	• Sample was not optimal for both internal validity and representativeness<br>• Subjects with thyroid disease were not explicitly excluded[1]<br>• Relevant confounders not controlled for in statistical analysis<br>• Methodology for handling missing data not reported<br>• Effect modification analysis not conducted
Dineva et al. [45]<br>(all studies)[e]<br><br>Pregnant	5/9	Medium	• Sample was not optimal for both internal validity and representativeness<br>• Subjects with thyroid disease were not explicitly excluded[2]<br>• Assessment of the exposure was not valid for the study population<br>• Exposure was not characterized<br>• Effect modification analysis not conducted
Farha et al. [46]<br><br>Pregnant	4/9	Medium	• Sample was not optimal for both internal validity and representativeness<br>• Subjects with thyroid disease were not explicitly excluded[2]<br>• Assessment of the exposure was not valid for the study population<br>• Exposure was not characterized<br>• Relevant confounders not controlled for in statistical analysis<br>• Effect modification analysis not conducted
Ferreira et al. [47]<br><br>Pregnant	5/9	Medium	• Sample was not optimal for both internal validity and representativeness<br>• Subjects with thyroid disease were not explicitly excluded[2]<br>• Assessment of the exposure was not valid for the study population<br>• Relevant confounders not controlled for in statistical analysis<br>• Methodology for handling missing data not reported

[1] Thyroid disease was not an exclusion criterion

[2] Subjects with thyroid disease at baseline were excluded; however, the development of thyroid disease during the study was not assessed

**Reference**<br>**Study Population**	**Quality Score**	**Quality Rating[a]**	**Key Limitations[b]**
González-Martínez et al. [49]<br><br>Pregnant	4/9	Medium	• Sample was not optimal for both internal validity and representativeness<br>• Subjects with thyroid disease were not explicitly excluded[1]<br>• Assessment of the exposure was not valid for the study population<br>• Exposure was not characterized<br>• Methodology for handling missing data not reported<br>• Effect modification analysis not conducted
Gunnarsdottir et al. [50]<br><br>Pregnant	4/9	Medium	• Sample was not optimal for both internal validity and representativeness<br>• Subjects with thyroid disease were not explicitly excluded[2]<br>• Assessment of the exposure was not valid for the study population<br>• Exposure was not characterized<br>• Relevant confounders not controlled for in statistical analysis<br>• Effect modification analysis not conducted
Henjum et al. [51]<br><br>Lactating	3/9	Low	• Sample was not optimal for both internal validity and representativeness<br>• Subjects with thyroid disease were not explicitly excluded[2]<br>• Assessment of the exposure was not valid for the study population<br>• Characterization of exposure was not optimal<br>• Relevant confounders not controlled for in statistical analysis<br>• Methodology for handling missing data not reported<br>• Effect modification analysis not conducted
Huang et al. [52]<br><br>Pregnant	5/9	Medium	• Sample was not optimal for both internal validity and representativeness<br>• Subjects with thyroid disease were not explicitly excluded[2]<br>• Assessment of the exposure was not valid for the study population<br>• Relevant confounders not controlled for in statistical analysis<br>• Effect modification analysis not conducted

[1] Thyroid disease was assessed during the study and all subjects were included, irrespective of thyroid disease status

[2] Thyroid disease was not an exclusion criterion.

Reference<br>Study Population	Quality Score	Quality Rating[a]	Key Limitations[b]
Huang et al. [53]<br><br>Lactating	4/9	Medium	• Sample was not optimal for both internal validity and representativeness<br>• Assessment of the exposure was not valid for the study population<br>• Characterization of exposure was not optimal<br>• Relevant confounders not controlled for in statistical analysis<br>• Effect modification analysis not conducted
Johannesen et al. [54]<br><br>Pregnant	4/9	Medium	• Sample was not optimal for both internal validity and representativeness<br>• Subjects with thyroid disease were not explicitly excluded[1]<br>• Assessment of the exposure was not valid for the study population<br>• Exposure was not characterized<br>• Relevant confounders not controlled for in statistical analysis<br>• Methodology for handling missing data not reported
Johnsen et al. [55]<br><br>Pregnant	3/9	Low	• Sample was not optimal for both internal validity and representativeness<br>• Subjects with thyroid disease were not explicitly excluded[2]<br>• Assessment of the exposure was not valid for the study population<br>• Exposure was not characterized<br>• Reporting of results in accordance with a protocol could not be determined<br>• Methodology for handling missing data not reported<br>• Effect modification analysis not conducted
Jorgensen et al. [56]<br><br>Lactating	5/9	Medium	• Sample was not optimal for both internal validity and representativeness<br>• Subjects with thyroid disease were not explicitly excluded[2]<br>• Validity of the exposure assessment cannot be determined<br>• Characterization of exposure was not optimal<br>• Effect modification analysis not conducted

[1] Thyroid disease was not an exclusion criterion.

[2] Subjects with thyroid disease at baseline were excluded; however, the development of thyroid disease during the study was not assessed.

Reference<br>Study Population	Quality Score	Quality Rating[a]	Key Limitations[b]
Kedir et al. [57]<br><br>Pregnant	4/9	Medium	• Sample was not optimal for both internal validity and representativeness<br>• Subjects with thyroid disease were not explicitly excluded[1]<br>• Assessment of the exposure was not valid for the study population<br>• Exposure was not characterized<br>• Methodology for handling missing data not reported<br>• Effect modification analysis not conducted
Knight et al. [58]<br><br>Pregnant	5/9	Medium	• Sample was not optimal for both internal validity and representativeness<br>• Assessment of the exposure was not valid for the study population<br>• Relevant confounders not controlled for in statistical analysis<br>• Effect modification analysis not conducted
Lean et al. [59]<br><br>Pregnant	4/9	Medium	• Sample was not optimal for both internal validity and representativeness<br>• Subjects with thyroid disease were not explicitly excluded[2]<br>• Assessment of the exposure was not valid for the study population<br>• Exposure was not characterized<br>• Relevant confounders not controlled for in statistical analysis<br>• Methodology for handling missing data not reported
Liu et al. [60]<br><br>Pregnant	5/9	Medium	• Sample was not optimal for both internal validity and representativeness<br>• Assessment of the exposure was not valid for the study population<br>• Exposure was not characterized<br>• Effect modification analysis not conducted
Melero et al. [62]<br><br>Pregnant	5/9	Medium	• Sample was not optimal for both internal validity and representativeness<br>• Subjects with thyroid disease were not explicitly excluded[3]<br>• Assessment of the exposure was not valid for the study population<br>• Relevant confounders not controlled for in statistical analysis<br>• Effect modification analysis not conducted

[1] Thyroid disease was not an exclusion criterion.

[2] Subjects with thyroid disease at baseline were excluded; however, the development of thyroid disease during the study was not assessed.

[3] Thyroid disease was assessed during the study and all subjects were included, irrespective of thyroid disease status.

Reference<br>Study Population	Quality Score	Quality Rating[a]	Key Limitations[b]
Menéndez Torre et al. [63]<br><br>Pregnant	5/9	Medium	• Sample was not optimal for both internal validity and representativeness<br>• Subjects with thyroid disease were not explicitly excluded[1]<br>• Validity of the exposure assessment cannot be determined<br>• Relevant confounders not controlled for in statistical analysis<br>• Effect modification analysis not conducted
Mian et al. [64]<br><br>Pregnant	3/9	Low	• Sample was not optimal for both internal validity and representativeness<br>• Subjects with thyroid disease were not explicitly excluded[2]<br>• Assessment of the exposure was not valid for the study population<br>• Exposure was not characterized<br>• Conflict of interest statement not provided<br>• Relevant confounders not controlled for in statistical analysis<br>• Effect modification analysis not conducted
Moon et al. [65]<br><br>Lactating	4/9	Medium	• Sample was not optimal for both internal validity and representativeness<br>• Subjects with thyroid disease were not explicitly excluded[1]<br>• Assessment of the exposure was not valid for the study population<br>• Conflict of interest statement not provided<br>• Relevant confounders not controlled for in statistical analysis<br>• Methodology for handling missing data not reported
Ollero et al. [67]<br><br>Pregnant	4/9	Medium	• Sample was not optimal for both internal validity and representativeness<br>• Subjects with thyroid disease were not explicitly excluded[3]<br>• Validity of the exposure assessment cannot be determined<br>• Relevant confounders not controlled for in statistical analysis<br>• Methodology for handling missing data not reported<br>• Effect modification analysis not conducted

[1] Thyroid disease was not an exclusion criterion.

[2] Subjects with thyroid disease at baseline were excluded; however, the development of thyroid disease during the study was not assessed.

[3] Thyroid disease was assessed during the study and all subjects were included, irrespective of thyroid disease status.

Reference<br>Study Population	Quality Score	Quality Rating[a]	Key Limitations[b]
Opazo et al. [29]<br><br>Pregnant	6/9	High	• Assessment of the exposure was not valid for the study population<br>• Relevant confounders not controlled for in statistical analysis<br>• Effect modification analysis not conducted
Perrine et al. [68]<br><br>Pregnant	5/9	Medium	• Sample was not optimal for both internal validity and representativeness<br>• Subjects with thyroid disease were not explicitly excluded[1]<br>• Assessment of the exposure was not valid for the study population<br>• Exposure was not characterized<br>• Effect modification analysis not conducted
Petersen et al. [69]<br><br>Lactating	3/9	Low	• Sample was not optimal for both internal validity and representativeness<br>• Subjects with thyroid disease were not explicitly excluded[2]<br>• Assessment of the exposure was not valid for the study population<br>• Characterization of exposure was not optimal<br>• Relevant confounders not controlled for in statistical analysis<br>• Methodology for handling missing data not reported<br>• Effect modification analysis not conducted
Prpić et al. [70]<br><br>Lactating	3/9	Low	• **Sample was not optimal for both internal validity and representativeness**[f]<br>• Assessment of the exposure was not valid for the study population<br>• Assessment of the outcome was not valid<br>• Conflict of interest statement not provided<br>• Relevant confounders not controlled for in statistical analysis<br>• Effect modification analysis not conducted

[1] Subjects with thyroid disease at baseline were excluded; however, the development of thyroid disease during the study was not assessed.

[2] Thyroid disease was not an exclusion criterion.

Reference<br>Study Population	Quality Score	Quality Rating[a]	Key Limitations[b]
Refaat et al. [71]<br><br>Pregnant	4/9	Medium	• Sample was not optimal for both internal validity and representativeness<br>• Subjects with thyroid disease were not explicitly excluded[1]<br>• Validity of the exposure assessment cannot be determined<br>• Exposure was not characterized<br>• Relevant confounders not controlled for in statistical analysis<br>• Effect modification analysis not conducted
Silva et al. [72]<br><br>Pregnant	3/9	Low	• Sample was not optimal for both internal validity and representativeness<br>• Subjects with thyroid disease were not explicitly excluded[2]<br>• Assessment of the exposure was not valid for the study population<br>• Exposure was not characterized<br>• Assessment of the outcome was not valid<br>• Relevant confounders not controlled for in statistical analysis<br>• Effect modification analysis not conducted
Stråvik et al. [73]<br><br>Pregnant/lactating	3/9	Low	• Sample was not optimal for both internal validity and representativeness<br>• Subjects with thyroid disease were not explicitly excluded2<br>• Assessment of the exposure was not valid for the study population<br>• Exposure was not characterized<br>• Relevant confounders not controlled for in statistical analysis<br>• Methodology for handling missing data not reported<br>• Effect modification analysis not conducted
Torres et al. [76]<br><br>Pregnant	6/9	High	• Sample was not optimal for both internal validity and representativeness<br>• Subjects with thyroid disease were not explicitly excluded2<br>• Relevant confounders not controlled for in statistical analysis<br>• Effect modification analysis not conducted

[1] Thyroid disease was assessed during the study and all subjects were included, irrespective of thyroid disease status.

[2] Subjects with thyroid disease at baseline were excluded; however, the development of thyroid disease during the study was not assessed.

Reference<br>Study Population	Quality Score	Quality Rating[a]	Key Limitations[b]
Trabzuni et al. [77]<br><br>Lactating	3/9	Low	• Sample was not optimal for both internal validity and representativeness<br>• Subjects with thyroid disease were not explicitly excluded[1]<br>• Validity of the exposure assessment cannot be determined<br>• Characterization of exposure was not optimal<br>• Conflict of interest statement not provided<br>• Relevant confounders not controlled for in statistical analysis<br>• Effect modification analysis not conducted
Vandevijvere et al. [78]<br><br>Pregnant	5/9	Medium	• Sample was not optimal for both internal validity and representativeness<br>• Subjects with thyroid disease were not explicitly excluded[1]<br>• Validity of the exposure assessment cannot be determined<br>• Methodology for handling missing data not reported<br>• Effect modification analysis not conducted
Veisa et al. [79]<br><br>Pregnant	5/9	Medium	• Sample was not optimal for both internal validity and representativeness<br>• Subjects with thyroid disease were not explicitly excluded[2]<br>• Validity of the exposure assessment cannot be determined<br>• Relevant confounders not controlled for in statistical analysis<br>• Effect modification analysis not conducted
Wu et al. [80]<br><br>Pregnant	2/9	Low	• Sample was not optimal for both internal validity and representativeness<br>• Subjects with thyroid disease were not explicitly excluded[2]<br>• Validity of the exposure assessment cannot be determined<br>• Exposure was not characterized<br>• Assessment of the outcome was not valid<br>• Conflict of interest statement not provided<br>• Relevant confounders not controlled for in statistical analysis<br>• Effect modification analysis not conducted

[1] Thyroid disease was not an exclusion criterion.

[2] Subjects with thyroid disease at baseline were excluded; however, the development of thyroid disease during the study was not assessed.

**Reference**<br>**Study Population**	**Quality Score**	**Quality Rating**[a]	**Key Limitations**[b]
Zhao et al. [75]<br><br>Pregnant	3/9	Low	• Sample was not optimal for both internal validity and representativeness<br>• Subjects with thyroid disease were not explicitly excluded[1]<br>• Validity of the exposure assessment cannot be determined<br>• Assessment of the outcome was not valid<br>• Relevant confounders not controlled for in statistical analysis<br>• Methodology for handling missing data not reported<br>• Effect modification analysis not conducted
**Assessment of Bath et al. [35] using the OSQE tool**[g]			
Bath et al. [35]<br><br>Pregnant	CS: 6/9<br><br>PC: 10/15	CS: High<br><br>PC: High	**Limitations in both assessments:**<br>• Sample was not optimal for both internal validity and representativeness<br>• Subjects with thyroid disease were not explicitly excluded[2]<br>• Assessment of the exposure was not valid for the study population<br>• Methodology for handling missing data not reported<br>**Limitations as a prospective cohort study:**<br>• Assessor was not blinded to the exposure<br>• Exclusion of subjects with the outcome at baseline not reported

[1] Thyroid disease was not an exclusion criterion.

[2] Subjects with thyroid disease at baseline were excluded; however, the development of thyroid disease during the study was not assessed.

Reference<br>Study Population	Quality Score	Quality Rating[a]	Key Limitations[b]
**Assessment of RCT using RoB2 tool[h]**			
Nazeri et al. [66]<br><br>Lactating	NA	Risk of Bias: High	• Lack of allocation concealment (high risk of bias)<br>• Could not determine selective reporting due to lack of prespecified statistical analysis plan (some concerns)

CS = cross-sectional; OSQE = Observational Study Quality Evaluation; PC = prospective cohort; RCT = randomized controlled trial; RoB2 = Risk of Bias 2.

[a] The quality of observational studies was assessed using the OSQE tool. The maximum possible score for the OSQE cohort study tool was 15. Cohort studies with a score ≤7 were classified as low quality, a score of 8 as medium quality, and a score ≥9 as high quality. The maximum possible score for the OSQE cross-sectional study tool was 9. Cross-sectional studies with a score of 2 to 3 were classified as low quality, 4 to 5 as medium quality, and ≥6 as high quality. It should be noted that any study wherein 1 or more veto criteria were met, the affected criterion did not receive a score. Quality classification thresholds were set by group consensus. The quality of randomized controlled trials was assessed using the Cochrane RoB2 tool. If a study had 1 or more domains rated as "some concerns" or any domain rated as "high risk," the overall quality was classified as high risk of bias.

[b] Veto criterion are bolded.

[c] Study designs were determined based on the relevant analyses reported in the study. As such, a study where relevant outcomes were reported cross-sectionally were appraised using the cross-sectional version of the OSQE tool, even if the study cohort itself was a prospective cohort.

[d] Overlapping tertiles for dairy product intake per week.

[e] The publication by Dineva et al. [45] reported results from 3 observational studies (Avon Longitudinal Study of Parents and Children; Generation R cohort; INfancia y Medio Ambiente). The study quality appraisal is applicable for all 3 studies.

[f] The study population was not reflective of the general population as all recruited women had a university degree.

[g] In the study by Bath et al. [35], the study was appraised using both tools given that cross-sectional and prospective cohort data reported within the publication were considered relevant for this review.

[h] RCTs appraised using the RoB2 tool were rated to either have low or high risk of bias or some concerns based on the algorithm provided by Sterne et al. [23].
