# Supplementary material for: Dairy Intake and Iodine Status in Pregnant and Lactating Women: A Systematic Review and Meta-Analysis
Source: Nutrients. 2025 Nov 30;17(23):3765. doi: 10.3390/nu17233765 (PMC12693841; doi:10.3390/nu17233765)
Supplement: Supplementary file 1 [file nutrients-17-03765-s001.zip › Table S6_DMI_GRADEPro 25Nov2025.pdf]

Supplementary Table S6. GRADEPro assessment of iodine status outcomes in relation to dairy intake during pregnancy and lactation.

Certainty Assessment							No. of Patients		Effect		Certainty
**No. of Studies**	**Study Design**	**Risk of Bias**	**Inconsistency**	**Indirectness**	**Imprecision**	**Other Considerations**	**High Dairy Intake**	**Low Dairy Intake**	**Relative (95% CI)**	**Absolute (95% CI)**	
**Urinary iodine status (based on mean and SD data) (assessed with: spot urine and laboratory tests)**											
23	Non-randomized studies	Serious[a]	Serious[b]	Not serious	Not serious	All plausible residual confounding would suggest spurious effect, while no effect was observed dose response gradient	3923	2927	-	SMD **0.326 SD higher** (0.228 higher to 0.424 higher)	⊕⊕◯◯ Low[a,b]
**Iodine deficiency (assessed with: urinary biomarkers and laboratory tests)**											
11	Non-randomized studies	Serious[a]	Not serious	Not serious	Not serious	All plausible residual confounding would suggest spurious effect, while no effect was observed dose response gradient	638/1027 (62.1%)[c]	690/1010 (68.3%)[c]	**OR 0.581** (0.484 to 0.698)	**127 fewer per 1000** (from 172 fewer to 82 fewer)	⊕⊕⊕◯ Moderate[a]
**Urinary iodine status (based on Fisher's z from β-coefficients) (assessed with: spot urine and laboratory tests)**											
10	Non-randomized studies	Serious[a]	Serious[b]	Not serious	Not serious	All plausible residual confounding would suggest spurious effect, while no effect was observed dose response gradient	[d]	[d]	-	SMD **0.228 SD higher** (0.154 higher to 0.301 higher)	⊕⊕◯◯ Low[a,b]
**Urinary iodine status (based on correlations) (assessed with: spot urine and laboratory tests)**											
2	Non-randomized studies	Serious[a]	Very serious[e]	Not serious	Serious[f]	All plausible residual confounding would suggest spurious effect, while no effect was observed dose response gradient	[h]	[h]	-	SMD **0.416 SD higher** (0.088 lower to 0.919 higher)	⊕◯◯◯ Very low[a,e,f]

Certainty Assessment							No. of Patients		Effect		Certainty
No. of Studies	Study Design	Risk of Bias	Inconsistency	Indirectness	Imprecision	Other Considerations	High Dairy Intake	Low Dairy Intake	Relative (95% CI)	Absolute (95% CI)	
**Iodine deficiency (assessed with: dietary intake measures)**											
2	Non-randomized studies	Serious[a]	Very serious[e]	Not serious	Not serious	Very strong association all plausible residual confounding would suggest spurious effect, while no effect was observed dose response gradient	-/45,602[i]	-/18,826	**OR 0.060** (0.010 to 0.381)	**0 fewer per 1000** (from 0 fewer to 0 fewer)	⊕⊕⊕◯ Moderate[a,e]
**Iodine intake through foods (based on mean and SD data) (assessed with: dietary intake measures)**											
2	Non-randomized studies	Serious[a]	Not serious	Not serious	Not serious	All plausible residual confounding would suggest spurious effect, while no effect was observed dose response gradient[j]	31,350	17,201	-	SMD **0.924 SD higher** (0.794 higher to 1.053 higher)[k]	⊕⊕⊕◯ Moderate[a,j]
**Breast milk iodine concentration (based on mean and SD) (assessed with: spot breast milk and laboratory tests)**											
2	Non-randomized studies	Very serious[l]	Very serious[e]	Not serious	Very serious[f]	All plausible residual confounding would suggest spurious effect, while no effect was observed dose response gradient	[m]	[m]	-	SMD **0.247 SD higher** (0.703 lower to 1.198 higher)	⊕◯◯◯ Very low[e,f,l]

CI = confidence interval; MD = mean difference; OR = odds ratio; OSQE = Observational Study Quality Evaluation; SD = standard deviation; SMD = standardized mean difference.

[a] Downgraded 1 level for risk of bias, as the majority of included studies were rated as medium quality, based on the OSQE tool for observational studies.

[b] Downgraded 1 level due to high heterogeneity (inconsistency).

[c] Three studies reported component-specific contrasts (different dairy types including milk, cheese, and yogurt) with potential participant overlap; to avoid double counting, these studies were included in the relative effect but excluded from the summed events/totals used for absolute effects. One study reported only an OR (no group-specific counts). Absolute effects are therefore based on studies (n=7) with a single non-overlapping contrast. The pooled OR (0.581; 95% CI 0.484, 0.698) includes all studies.

[d] Data entered as Fisher's Z transformed from beta (continuous association); exposed/non-exposed group counts were not applicable.

[e] Downgraded 2 levels due to very high heterogeneity (inconsistency).

[f] Downgraded 1 level for imprecision due to the pooled 95% CI crossing the line of no effect.

[g] Events/totals could not be summed across all studies. One study reported only OR (no group counts), and another study reported component-specific contrasts (milk and yogurt) with potential participant overlap. To avoid double counting and bias from a single small study, raw counts were not used.

[h] Data entered as correlation coefficients; exposed/non-exposed group counts were not applicable.

[i] Events were not entered because 1 contributing study did not report group-specific counts. To avoid a biased baseline from the smaller study, absolute effects were not derived from raw counts. The relative effect and total participants reflect both studies.

[j] We did not apply large effect since evidence is limited to two heterogeneous studies ($I^2 \approx 81\%$).

[k] Primary analysis used mean difference (same units across studies) with random effects. Heterogeneity was high ($I^2 = 80.7\%$) due to only 2 studies and extreme imbalance in sample sizes; we report MD for interpretability

[l] Downgraded 2 levels for risk of bias, as all the studies were rated as low quality, based on the OSQE tool for observational studies

[m] Data entered as correlation measures; exposed/non-exposed group counts were not applicable.
