# Supplementary material for: Dairy Intake and Iodine Status in Pregnant and Lactating Women: A Systematic Review and Meta-Analysis
Source: Nutrients. 2025 Nov 30;17(23):3765. doi: 10.3390/nu17233765 (PMC12693841; doi:10.3390/nu17233765)
Supplement: Supplementary file 1 [file nutrients-17-03765-s001.zip › Table S7_DMI_other MA and Subgroup analyses (dairy & iodine) 25Nov2025.pdf]

Supplementary Table S7. Meta-analysis showing the association between dairy intake and different iodine status outcomes[a]

Outcomes	Meta-Analysis				Heterogeneity		
	**Study Group**	**Number of Cohorts**	**Effect Estimate (95% CI)**[b]	***P* Effect**	**Q Statistic**	***P* Heterogeneity**	***I*²** **(%)**
Urinary iodine status (beta coefficients and correlations, converted to Fisher's Z) in relation to dairy intake[b]	Overall	10	0.228 (0.154, 0.301)	<0.001	22.219	0.008	59.495
	**Adjustment status**						
	Adjusted	7	0.243 (0.145, 0.340)	<0.001	12.162	0.058	50.667
	Unadjusted (crude estimates)	3	0.203 (0.07, 0.336)	0.003	9.743	0.008	79.473
	**Data based on correlation coefficients**[c]						
	Overall	2	0.416 (−0.08, 0.919)	0.106	4.311	0.038	76.803
Dietary iodine deficiency in relation to dairy intake[d]	Overall (adjusted data)	2	0.06 (0.01, 0.381)	0.003	296.238	<0.001	99.662
	Overall (unadjusted data)	2	0.081 (0.023, 0.287)	<0.001	150.205	<0.001	99.334
Breast milk iodine in relation to dairy intake[b]	Overall	2	0.247 (-0.703, 1.198)	0.61	9.518	0.002	89.494
Dietary iodine intake in relation to dairy intake[b]	Overall	2	0.924 (0.794, 1.053)	<0.001	1.052	0.305	4.936

CI = confidence interval.

[a] Main analysis was conducted using random-effects model. Subgroup analyses were conducted using mixed-effects model.

[b] The effect estimate represents the pooled standardized mean difference.

[c] Individual random-effects analyses were performed including relative studies.

[d] The effect estimate represents the odd ratio of iodine deficiency.
